# Extracting physical characteristics of higher-order chromatin structures from 3D image data

**DOI:** 10.1016/j.csbj.2022.06.018

**Published:** 2022-06-20

**Authors:** William Franz Lamberti, Chongzhi Zang

**Affiliations:** a Center for Public Health Genomics, University of Virginia, Charlottesville, VA 22908, USA; b Department of Public Health Sciences, University of Virginia, Charlottesville, VA 22908, USA

**Keywords:** 3D super-resolution imaging, Chromatin structure, Physical characteristics, Spatial genomics

## Abstract

Higher-order chromatin structures have functional impacts on gene regulation and cell identity determination. Using high-throughput sequencing (HTS)-based methods like Hi-C, active or inactive compartments and open or closed topologically associating domain (TAD) structures can be identified on a cell population level. Recently developed high-resolution three-dimensional (3D) molecular imaging techniques such as 3D electron microscopy with in situ hybridization (3D-EMSIH) and 3D structured illumination microscopy (3D-SIM) enable direct detection of physical representations of chromatin structures in a single cell. However, computational analysis of 3D image data with explainability and interpretability on functional characteristics of chromatin structures is still challenging. We developed Extracting Physical-Characteristics from Images of Chromatin Structures (EPICS), a machine-learning based computational method for processing high-resolution chromatin 3D image data. Using EPICS on images produced by 3D-EMISH or 3D-SIM techniques, we generated more direct 3D representations of higher-order chromatin structures, identified major chromatin domains, and determined the open or closed status of each domain. We identified several high-contributing features from the model as the major physical characteristics that define the open or closed chromatin domains, demonstrating the explainability and interpretability of EPICS. EPICS can be applied to the analysis of other high-resolution 3D molecular imaging data for spatial genomics studies. The R and Python codes of EPICS are available at https://github.com/zang-lab/epics.

## Graphical abstract

**Figure:**
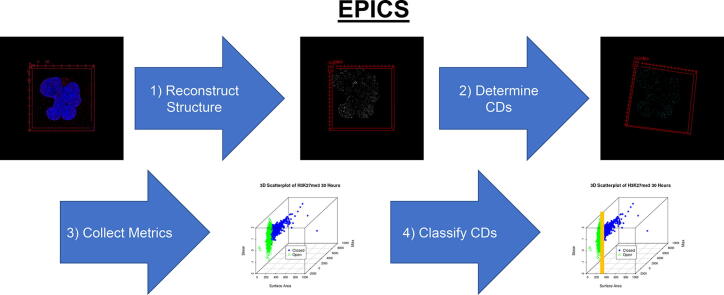

## 1 Introduction

The genomic DNA is packaged into chromatin in a hierarchical structure in the eukaryotic cell nucleus. The higher-order chromatin structure has major impacts on gene regulation, cell identity, and human health [1], [2]. The fundamental unit of these structures are nucleosomes, DNA wrapped around a histone octamer core. The string of nucleosomes form various dynamic structures that can be characterized into domains such as topologically associating domains (TADs) [3]. Higher in the hierarchy, TADs can be categorized into compartment structures which can be defined as active (open) or inactive (closed). Further on the hierarchy, efforts have been made to use genome-scale imaging to map chromatin structure at a genome scale within a single cell while providing nuclear speckles and nucleoli [4].

Open and closed chromatin have important biological roles. For instance, there is evidence that subtypes of cancers have different gene expression patterns associated with open and closed TADs [5], [6]. The structural properties of genome organization can be measured using high-throughput sequencing (HTS)-based technologies, like Hi-C [7], [8] and ChIA-PET [9], which primarily use DNA sequences as a positioning and quantification tool to determine the average proximity between two regions in the genome accumulated from a population of cells [7], [10]. Although the 3D configuration of chromatin can be inferred from Hi-C data using statistical or computational models [11], [12], direct measurement for physical characteristics of 3D chromatin structure in a single cell is still challenging [13].

Computer simulations of genetic structures have replicated and advanced our understanding of chromatin structures [14]. For example, MiChroM-based technologies are able to simulate chromatin structures and approximate Hi-C contact maps [15], [16], [17]. The resulting simulation pipelines have been compared to fluorescence in situ hybridization (FISH)-based approaches [18]. The Nucleome Data Bank is a resource that simulates chromatin structures using MiChroM [19]. However, obtaining spatial results based on more direct imaging-based technologies is critical to ensure that simulations match the true structures.

Recent development of spatial omics technologies enables high-resolution detection of spatial distributions of molecular genomic information such as gene expression and genome organization by applying HTS techniques, such as 10X Visium [20] and Slide-seq [21], or using super-resolution microscopy techniques, such as seqFISH [22] and MERFISH [23]. Super-resolution microscopy has also been applied to measure chromatin structures directly, with emerged techniques such as 3D assay for transposase-accessible chromatin-photoactivated localization microscopy (ATAC-PALM) [24], 3D electron microscopy with in situ hybridization (3D-EMISH) [13], and 3D structured illumination microscopy (3D-SIM) [25]. ATAC-PALM is able to image the accessible genome at the nanometer scale and in conjunction with FISH-based techniques. 3D-EMISH is able to extract structures of probed genomic regions of interest and describe the domain structures at the nanometer scale. 3D-SIM is able to visualize chromatin throughout the cell with a 39.5 nm resolution. Furthermore, FISH-based chromatin imaging techniques such as ORCA [1], it’s predecessor [26], and MINA [27] have generated higher-order chromatin structures including TADs and A/B compartments consistent with what have been inferred from Hi-C, suggesting that chromatin domains (CDs) reconstructed from image data should reveal the same biological functions [25]. Compared with Hi-C, imaging-based methods provide more direct measurements of physical representations of higher-order chromatin structures directly in a single cell [13]. However, computational analysis of 3D chromatin image data remains a challenge. Specifically, computational models to connect active or inactive chromatin domains with the physical characteristics from 3D images are essentially nonexistent. While others have found statistically significant features (surface area, volume, and sphercity) to discriminate between discovered clusters that tend to associate with active and inactive chromatin [28], a formalized model combining these and other terms has not been reported. Additionally, these computational approaches need to be developed while considering explainability and interpretability [29], so that biological insights can be generated from computational studies. Creating a computational model where scientists cannot ascertain the biological meaning is less useful than a computational model which can provide these insights. Thus, utilizing a machine learning (ML) approach which is easily interpretable and explainable is key for understanding the complex nature of 3D image data of chromatin.

In recent years, complex computational systems and advanced ML-based artificial intelligence (AI) have penetrated numerous fields of study [30]. Explainable AI (XAI) aims to provide explainable and interpretable insights to scientific inquiries [29], [31], [32]. Using XAI in biology means that the models and parameters can describe biological phenomena and characterize biological entities of interest in an explainable and interpretable fashion. Ensuring that the tenants of explainability and interpretability are met allows for scientists to evaluate if a computational model is adding to scientific knowledge. This work is built upon our previous model development work for extracting shape features from 2D image data such as blood cells [33] and satellite image classification [34], which adheres to the concepts of explainability and interpretability. Following the same principles, we extend our work for modeling 3D image data focusing on chromatin structure and to characterize 3D chromatin domains as open (active) and closed (inactive). This method allows for scientists to provide clear biological insights to various and dynamic structures of chromatin and their physical characteristics.

In this paper, we present Extracting Physical-Characteristics from Images of Chromatin Structures (EPICS), a method able to characterize different chromatin domains from 3D image data generated from two techniques: 3D-EMISH and 3D-SIM. We apply EPICS to 3D-EMISH and 3D-SIM image data to characterize open or closed chromatin domains from each data type. We then interpret the results of EPICS by identifying the most important physical characteristics as features that distinguish chromatin domains for biological insights. Our work provides a spatial and physical perspective of 3D image data modeling for functional genomics.

## 2 Materials and methods

**Fig. 1:**
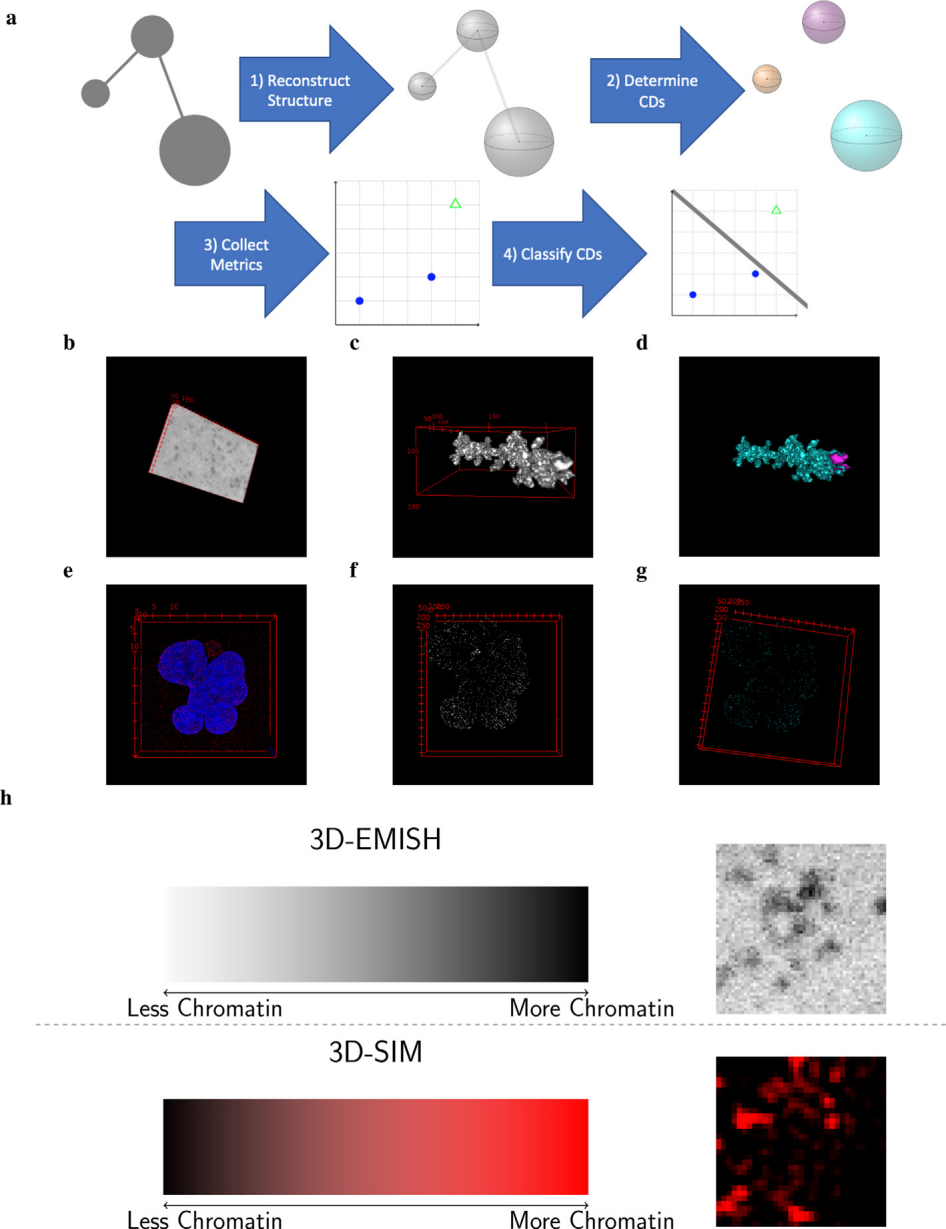
Schematic of EPICS with examples of images. **(a)** Schematic of EPICS. The gray circles represent the raw imaging data’s potential chromatin domains (CDs). The gray spheres represent reconstructed CDs. Each colorized sphere represents a uniquely identified CD (three in this case). The blue dots and green open triangle represent the closed and open CDs in the feature space. The line separating the points in the feature space represents the created model to classify the closed and open CDs from one another. The steps in **(a)** are exemplified with 3D-EMISH data in **b** - **d** and the 3D-SIM data in **e** - **g**. **(b)** Example of the raw input image from 3D-EMISH. **(c)** The reconstructed structure of the chromatin object of interest. **(d)** The resulting CDs. Each color represents a unique CD. In this case, there are two CDs with a larger cyan CD and a smaller pink CD. **(e)** The DAPI and H3K27me3 3D-SIM raw image data for the 30 hour treatment. **(f)** The reconstructed structure of the chromatin object of interest. **(g)** All identified CDs from the 30 hour treatment image. Each shade of cyan represents a unique CD. In this case, there are hundreds of different CDs present. The animated .svg files of **b** - **g** are provided at our GitHub link. **(h)** A color bar for each technologies raw pixel values. Examples of a cropped section of a slice from a 3D image is provided next to each graphical description.

EPICS is a computational method we developed to characterize chromatin domains as open or closed from 3D image data. In this section, we first describe how EPICS reconstructs the chromatin from the raw image data. We then discuss our computational algorithm to determine if a chromatin domain is open or closed. This involves the selection of candidate metrics which are useful for determining if a chromatin domain is open or closed. It also involves identifying those variables which are the most important for classifying chromatin domains from one another. EPICS is summarized in Fig. 1a.

### 2.1 Defining chromatin domain assignment for images

We describe our solution for reconstructing the chromatin domain structure from the raw image data while adhering to the concepts of explainability and interpretability herein. Using image operator notation to represent the image processing operators applied to the input data [35], we first smooth the image via

(1) $$\left\{s[\overset{\to }{x}]\}=S\{t[\overset{\to }{x}]\right\}$$

where S is the smoothing operator, $\left\{t[\overset{\to }{x}]\right\}$ is the input image, and $\left\{s[\overset{\to }{x}]\right\}$ is the resulting smoothed image. We then isolate the relevant signals using

(2) $$\left\{i[\overset{\to }{x}]\}=L\{t[\overset{\to }{x}]\right\}$$

where L identifies the relevant signals of interest and $\left\{i[\overset{\to }{x}]\right\}$ is the set of images containing the isolated signals of interest. We then apply

(3) $$m[\overset{\to }{x}]=I_{B}i[\overset{\to }{x}]$$

where $I_{B}$ interpolates the object using the other slices to construct the missing slices and $m[\overset{\to }{x}]$ is the reconstructed object of interest from the given target signal image, $i[\overset{\to }{x}]$ (Fig. 1a, Step 1). Interpolation is necessary to ensure that each voxel is approximately a cube in physical space. We then determine the chromatin domains by

(4) $$\{d[\overset{\to }{x}]\}=Cm[\overset{\to }{x}],$$

where C determines the chromatin domains from the input image and $\left\{d[\overset{\to }{x}]\right\}$ are the set of resulting chromatin domains. The number of chromatin domains is then determined (Fig. 1a, Step 2). The chromatin domains are extracted from the 3D-EMISH and 3D-SIM data using Eqs. A32–A35 and A39–A40 respectively in the Supplementary Data.

We then collect a variety of explainable and interpretable metrics using the shorthand operator of D (Fig. 1a, Step 3):

(5) $$D=D\{d[\overset{\to }{x}]\}\text{.}$$

The equations for extract each metric are described in the Supplementary Data in Eqs. A13–A27. From these extracted metrics in our resulting matrix, D, we build a model that creates rules for predicting whether a particular chromatin domain is open or closed. In other words,

(6) $$f(D_{i})=\left\{\begin{array}{cc} A & \mathrm{Rule}\;1\\ B & \mathrm{Rule}\;2 \end{array}\right),\forall i,$$

where *i* is the $i^{\mathrm{th}}$ chromatin domain such that i∈{1,…,N},N is the total number of chromatin domains, and *A* and *B* are the two possible chromatin states, consistent with A/B compartments inferred from Hi-C data. A is considered active or open, while B is inactive or closed (Fig. 1a, Step 4).

This computational approach is similar in spirit to the computation required for A/B compartment assignment using data from Hi-C. EPICS and the computation using Hi-C data both use a set of rules to determine if a target is open (active) or closed (inactive). While Hi-C is based on correlations, EPICS uses a logistic regression model based on the physical characteristics of the objects. Supplementary Data A.3 provides an overview of the computation for A/B compartment assignment using Hi-C data. We provided this overview to help compare and contrast EPICS and Hi-C in a theoretical manner.

### 2.2 Defining open and closed domains for images

We need to identify potential candidate metrics that would be useful for clustering the chromatin domains and classifying the identified domains. To that end, we need to explicitly state what characterizes open and closed domains. Open domains have lots of space in between points and will be sparser. Closed domains are compact and close to one another. Natural choices for capturing these sparse and dense domains would be shape-based and intensity-based metrics. Shape metrics describe how dense the domain is spatially as indicated by Fig. B1. The intensity metrics describe the amount of the chromatin present in the sample. For 3D-EMISH data, low values indicate more object of interest, while larger values indicate that less object of interest is present (Fig. 1). To this end, we select 8 shape and 11 intensity metrics to describe our domains of interest, as summarized in Table 1.

**Table 1:** Metrics used in EPICS on a given chromatin domain, *i*. The first column is the $q^{\mathrm{th}}$ metric, where q∈{1,2,…,19}. The last columns correspond to the final estimated parameter for the logistic regression (LR) models for each data sets. ”-” refers to features not included in the model. ${\overset{\to }{m}}_{18}$ and ${\overset{\to }{m}}_{19}$ are not used for 3D-SIM data. The following symbol provided next to the coefficient value indicate different levels of significance: ***=0.001, **=0.01, *=0.05, .=0.1.

${\overset{\to }{m}}_{q,i}$	Metric	Type	3D-EMISH	3D-SIM		
				Control	6 Hrs	30 Hrs
-	Intercept	-	$94.282^{*\;*\;*}$	$85.408^{*\;*\;*}$	-6489	$73.738^{*\;*\;*}$
1	White EI	Shape	-	-	-	-
2	Black EI	Shape	-	-	-	-
3	SP	Shape	-	-	-	-
4	$1^{\mathrm{st}}$ Eigen.	Shape	$-0.001^{.}$	$0.094^{*}$	48.71	$0.801^{**}$
5	$2^{\mathrm{nd}}$ Eigen.	Shape	-	-	-	-
6	$3^{\mathrm{rd}}$ Eigen.	Shape	$-1.184^{*}$	-	-	$9.908^{.}$
7	Sphericity	Shape	$-8.906^{**}$	-	-	-
8	Surface Area	Shape	-	$0.065^{*\;*\;*}$	45.26	$0.364^{*\;*\;*}$
						
9	Mean	Intensity	-	-	-	-
10	SD	Intensity	-	-	1.639	$0.032^{**}$
11	Median	Intensity	-	-	-	-
12	Q1	Intensity	-	-	-	-
13	Q3	Intensity	$-0.001^{*\;*\;*}$	-	-	-
14	Max	Intensity	$-0.001^{*\;*\;*}$	$0.068^{*\;*\;*}$	1.487	$0.018^{*\;*\;*}$
15	Min	Intensity	-	$-0.015^{*}$	-	$-0.012^{**}$
16	Skewness	Intensity	-	-	294.0	$6.787^{*\;*\;*}$
17	Kurtosis	Intensity	-	-	-	-
18	% >24000	Intensity	-	-	-	-
19	% >30000	Intensity	-	-	-	-

The first shape metrics are the SPs and EIs, which are collected by extending shape proportion and encircled image-histogram (SPEI) algorithm [36] to be applicable to 3D shapes. The EI is the black and white pixel counts of the shape after the shape is placed in the minimum encompassing sphere and then the minimum encompassing cube. In other words, this is the volume and the surrounding volume of the object of interest. The SP value is the proportion of the volume of the shape relative to the sum of the EI. The other shape metrics collected that are used in the model are the eigenvalues of the shapes [35], sphericity [37], and surface area. The eigenvalues measure the major and minor axes of the shape. Sphericity measures how spherical a given shape is. Surface area is the 3D perimeter of the object of interest. This results in a total of 8 total shape metrics.

The intensity-based metrics are merely their respective statistic for the object’s intensity based values. For example, the mean of the intensity values measures the arithmetic mean of only the object’s voxel values. This does not include the background of the object. For the Mean, Median, Q1, Q3, Max, and Min Intensity metrics, low values indicate more or less chromatin for 3D-EMISH or 3D-SIM, respectively. High values indicate less or more chromatin for 3D-EMISH or 3D-SIM, respectively (Fig. 1). The remaining statistics are interpreted and explained in the typical manner. There are a total of 11 intensity-based metrics.

Extended explanations, interpretations, and image operators for each shape and intensity metric are provided in the Supplementary Data. In short, each of the metrics provided is explainable and interpretable. This helps to make the results of describing the open and closed chromatin domains explicitly understood and aid in understanding their biological underpinnings.

### 2.3 Determining rules for open-closed chromatin domains

Here we expand the notation from Eq. 6 to provide an explicit description of the analysis done in EPICS. For notation purposes, we have *J* batches such that j∈{1,2,…,J}. For the 3D-EMISH data, J=2. For the 3D-SIM experiments, J=1 since each experiment has a different treatment. Further, we have 19 features such that q∈{1,…,19}. Thus, ∀q,j, we perform

(7) |Dq,j|=Dq,j-μ^q,jσ^q,j,

where μ^q,j and σ^q,j are the sample mean and standard deviation of the $q^{\mathrm{th}}$ feature for the $j^{\mathrm{th}}$ batch. We do this to normalize the data and remove batch effects. We then perform the following to obtain the estimated A/B chromatin states:

(8) $${\overset{\to }{d}}_{,j}=K_{2}|D_{q,j}|$$

where K is the k-means clustering operator. In this case, we perform *k*-means clustering with a known number of clusters of 2. The output is the estimated chromatin states saved in an associated vector, ${\overset{\to }{d}}_{,j}$. Next, the average Mean intensity metric was used to automatically determine which cluster was open and closed. Specifically, we applied

(9) $$f({\overset{\to }{d}}_{,j})=\left\{\begin{array}{cc} m_{1} & M{\overset{\to }{m}}_{9,({\overset{\to }{d}}_{,j}==1)}\\ m_{2} & M{\overset{\to }{m}}_{9,({\overset{\to }{d}}_{,j}==2)} \end{array}\right)$$

This calculates the mean of of the Mean intensity metric for each cluster obtained by k-means. If large intensity values indicate more chromatin, then the rules are:

(10) $$f({\overset{\to }{d}}_{i,j}|m_{1}>m_{2})=\left\{\begin{array}{cc} A_{i,j} & {\overset{\to }{d}}_{,j}==2\\ B_{i,j} & {\overset{\to }{d}}_{,j}==1 \end{array}\right)$$

(11) $$f({\overset{\to }{d}}_{i,j}|m_{1}⩽m_{2})=\left\{\begin{array}{cc} A_{i,j} & {\overset{\to }{d}}_{,j}==1\\ B_{i,j} & {\overset{\to }{d}}_{,j}==2 \end{array}\right)$$

∀i. This is the rule applied for 3D-SIM. However, is small intensity values indicate more chromatin, then the rules are:

(12) $$f({\overset{\to }{d}}_{i,j}|m_{1}<m_{2})=\left\{\begin{array}{cc} A_{i,j} & {\overset{\to }{d}}_{,j}==2\\ B_{i,j} & {\overset{\to }{d}}_{,j}==1 \end{array}\right)$$

(13) $$f({\overset{\to }{d}}_{i,j}|m_{1}⩾m_{2})=\left\{\begin{array}{cc} A_{i,j} & {\overset{\to }{d}}_{,j}==1\\ B_{i,j} & {\overset{\to }{d}}_{,j}==2 \end{array}\right)$$

∀i. This is the pair of rules applied for 3D-EMISH.

However, the *k*-means clustering model does not provide any meaningful insight to which are the most important variables for discriminating the open and closed chromatin domains from one another. Thus, we select a logistic regression (LR) model to describe open and closed chromatin domains [38]. However, there are too many variables for this to be a truly interpretable and explainable model [29]. Thus, we use the least absolute shrinkage and selection operator (LASSO) algorithm to select the most important variables for our model [39], [40], [41]. Thus, we first model:

(14) $$\underset{\beta_{0},\beta }{\min}\frac{1}{N}\overset{N}{\underset{i=1}{\sum }}l(D_{i},d_{i},\alpha ,\beta ),$$

subject to $||\beta |{|}_{1}⩽\lambda$ where $||\cdot |{|}_{1}$ is the $L_{1}$-norm and λ is a tuning parameter [41]. The data was split into the training and validation data using a 70-30 split. We ensured that the proportions of the open and closed chromatin domains were preserved in the training and validation data using stratification [42]. We select the tuning parameter, λ, by using 10-fold cross validation on the training data. While the optimal λ is able to obtain a very high classification rate, it retains a larger number of variables. Thus, we select the λ value within 1 standard error for the 3D-EMISH data since it also has a very high classification rate, has less variables retained in the models, and is the more prudent choice (Supplementary Fig. B2). After repeating this process for the 3D-SIM, we chose the same value for the 3D-SIM’s choice of λ as the 3D-EMISH’s λ value to be more conservative [39], [40] (Supplementary Figs. B3–B5). Extended discussions on the choice of λ for the 3D-SIM data are provided in the Supplementary Data.

After we obtain the non-zero coefficients, we use those variables to model the following:

(15) $$\log\frac{P(C=B|D=x)}{P(C=A|D=x)}=\beta^{T}D\text{.}$$

The final estimates of the variables’ coefficients are found in the third through sixth columns of Table 1.

The parameters of the LR model are typically described using the log-odds ratio or the odds ratio [43], [38]. Thus we are able to interpret the log-odds ratio in the following manner for the $q^{\mathrm{th}}$ variable: assuming that all of the other variables are held constant, for every one unit increase for the given variable, we expect the log-odds of a being a closed chromatin domain to increase by β^q [43]. Positive values for estimated coefficients, β^q, and an increase in the associated variable corresponds to increasing the probability of being closed. Conversely, negative values for the estimated coefficient and an increase in the associated variable would indicate a decrease in the probability of being closed. Further, we are able to convert these to odds by taking the exponential of the estimated coefficient value.

For example, if we assume that all other variables do not change for the 3D-EMISH model, for every unit increase in the maximum of the intensity value of the chromatin domain, we expect the log-odds of being a closed chromatin domain to decrease by 0.001. The odds would be $e^{-0.001}=0.999$. Conversely, for the 3D-SIM image with the 6 hour treatment, we would expect the log-odds of a chromatin domain being closed to increase by 0.068. The odds would be $e^{0.068}=1.070$. This can be repeated for each variable of interest for each model.

This allows analysts to compare and contrast different models. Further, we are able to identify the similarities and differences between different data and treatments. For example, the 3D-SIM Control and 6 hours identify different parameters as being the most important. This suggests that there are tangible differences between the chromatin domains. In other words, we expect that the 6 hour treatment is fundamentally changing the chromatin structure. To quantify these difference, we can observe the parameter differences between the two models. For example, the max intensity parameter value differs by a factor of $\frac{1.487}{0.068}=21.87$. Thus, the 6 hour treatment impacts the max parameter value by about a factor of 21. Thus, this provides additional evidence that the treatment changes how chromatin domains exist in physical space.

Our EPICS method is considered XAI since they use features that are both interpretable and explainable: the LR model is a highly interpretable and explainable model to discriminate open and closed chromatin domains [29], and each operation performed during the entire process is clearly described while also being interpretable and explainable. The reduction in complexity is exemplified in Fig. B6.

### 2.4 Materials

Raw 3D-EMISH data was obtained from the Boettiger Lab’s GitHub ( https://github.com/3DEMISH/3D-EMISH) [1]. Raw 3D-SIM images were obtained from Cremer *et. al.*’s paper [25]. The computation was performed on a Ubuntu 18.04.5 system with 64 GB of RAM and an Intel®Xeon(R) W-2245 CPU @ 3.90GHz with 8 cores and 16 threads. For the image processing and metric collection, we used Python 3.6.9 [44] alongside numpy [45], scipy [46], skimage [47], sklearn [48], and kneed [49]. Determining the chromatin domain assignment was performed in R [50] using the xtable [51], caret [52], glmnet [53], and clue [54] packages.

## 3 Results

We applied EPICS on 3D chromatin image data from two techniques, 3D-EMISH (Fig. 1b–1d, Supplementary Figs. B7–B12) and 3D-SIM (Fig. 1, Fig. 1, Supplementary Figs. B13–B16). We identified open and closed chromatin domains from each data type, and explained and interpreted the model and results by extracting important physical characteristics from the image data. In addition, we identified batch effects from 3D-EMISH data and demonstrated that EPICS is able to characterize open and closed chromatin domains despite the batch effects.

### 3.1 Determination of open or closed chromatin domains from 3D-EMISH data

**Fig. 2:**
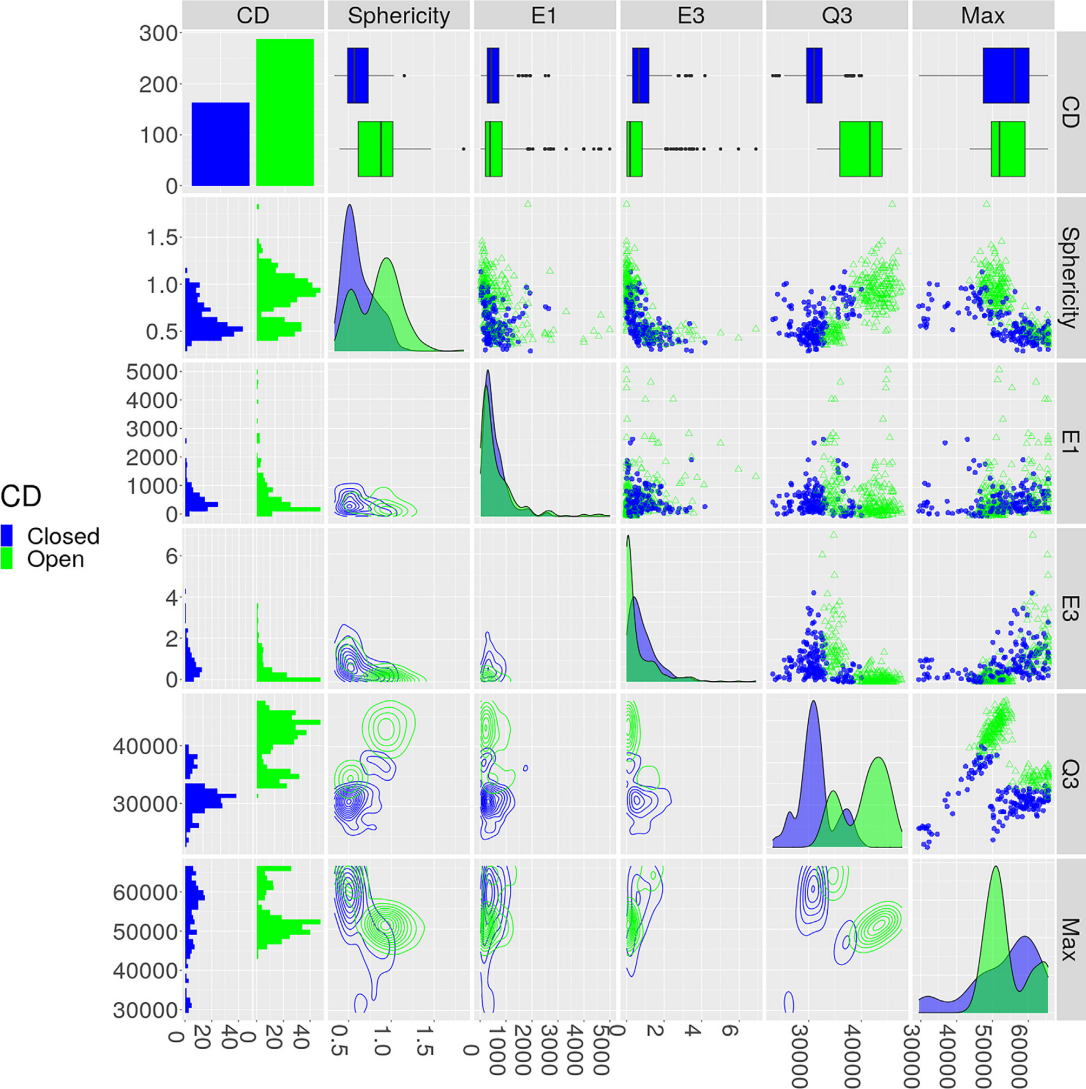
The important features for discriminating between open and closed chromatin domains (CDs) from the 3D-EMISH data. Open and closed CDs are green and blue dots, respectively. The top left cell provides the counts of closed and open CDs. The remaining cells in the top row provides a boxplot of each feature. The remaining first column provides a histogram of the closed and open CDs for each feature. The remaining diagonal cells provide the density plots of each feature by each class. The remaining upper triangular portion and bottom triangular portion provide the 2D scatterplots and the 2D contour plots, with corresponding axes labeled for each column and each row.

The 3D-EMISH data for our analysis is from Trazaskoma *et. al.* [13]. They analyzed human lymphoblastoid cells by probing a 1.7 mega-base (Mb) segment of the genome and extracted 229 image stacks or z-stacks of potential targets. Each voxel intensity value corresponds to a measured object as summarized in Fig. 1h. White intensities indicate less material, while darker colors correspond to more. Our computational analysis using EPICS determined out of 451 chromatin domains extracted from the 229 images, there exist 163 and 288 closed and open chromatin domains, respectively, across the two experiments. 19 shape and intensity-based measures of chromatin domains were used to determine these classes using *k*-means clustering. These initial clusters were verified using bootstrapped samples and evaluating them using Jaccard’s index (JI), accuracy, and balanced accuracy. All of the metrics indicated that the initial cluster found was stable and consistent across the replicates (Tables C1 and C2 and Figs. B17 and B18). We then used LASSO to select the appropriate features that are able to discriminate between the open and closed chromatin domains across the different batches (Fig. 2).

**Fig. 3:**
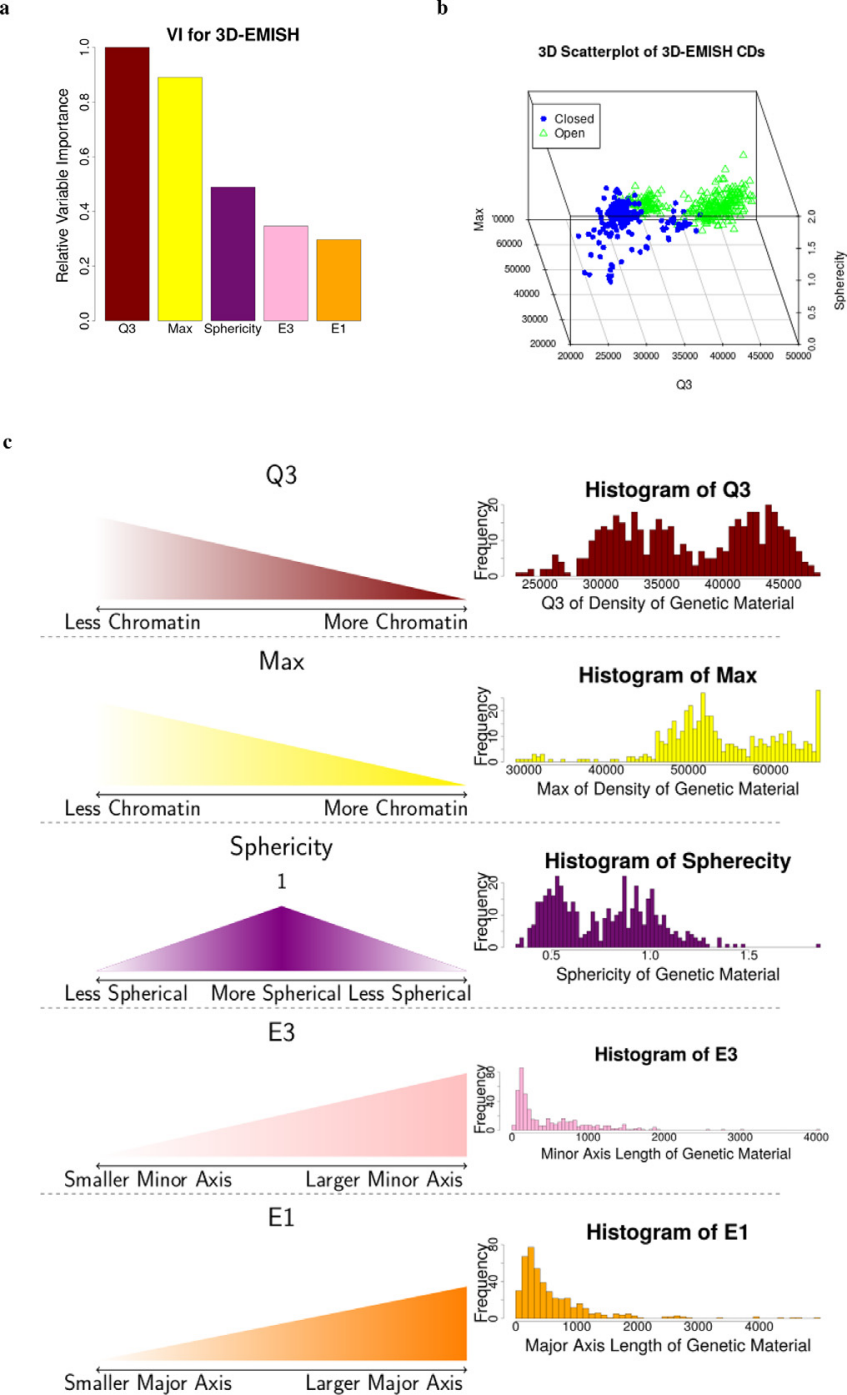
EPICS identified important biological features of chromatin domains from 3D-EMISH images. **(a)** Relative variable importance (VI) of the 5 most important features from the 19 candidate features. **(b)** The 3 most important variables in a 3D scatterplot. **(c)** Value interpretation and distribution of each identified important features for the 3D-EMISH data.

We then sorted these five variable from the most to least important using Variable Importance (VI). VI is the absolute value of each variables associated z-value from the LR model. Relative VI is VI divided by the largest z-value. The $3^{\mathrm{rd}}$ quantile (Q3) is the most important variable. The intensity-based metrics are the two most important variables, while the shape-based metrics are the three least important (Fig. 3a, Supplementary Table C3). Using the top three variables provides a clear separation in 3D space (Fig. 3b).

We identified five most important features to discriminate open and closed chromatin domains. Closed chromatin domains tend to have lower intensity values, as showcased by Q3. These lower intensity values indicate that more chromatin is present. This means that lower intensity values correspond to a denser object. Conversely, higher intensity values indicate that less chromatin is present. Thus, higher intensity values indicate that chromatin is more sparse or spread out. The maximum of the intensity of closed chromatin domains are smaller than those that are open. Thus, closed domains have more chromatin and are more densely packed than open domains. The sphericity of open domains tend to be closer to 1 than closed domains. Thus, open domains tend to be closer to a sphere in shape compared to closed domains. There are no obvious patterns for differentiating between open and closed chromatin domains when using the relative major and minor axis lengths (E1 and E3, respectively). However, alongside other metrics, their impact becomes more apparent (Fig. 2). All of these features have graphical representations provided in Fig. 3c. Thus, open domains tend to have less chromatin material and tend to be more spherical in shape, while closed domains tend to have more chromatin material and are less spherical in shape.

The logistic regression (LR) model created to discriminate open and closed chromatin domains using the 3D-EMISH data across the experiments was able to achieve an overall classification rate of about 95% (Table C4) and 96% (Table C5) on the training and validation data, respectively. Their associated 95% confidence intervals (CIs) for the overall classification rate were (92%, 97%) and (91%, 98%), respectively. Thus, our classification model performs well on data not used to build the model [29]. Further, since we used a LR model, our solution is highly interpretable and explainable [29].

### 3.2 Batch effects exist in 3D-EMISH data

**Fig. 4:**
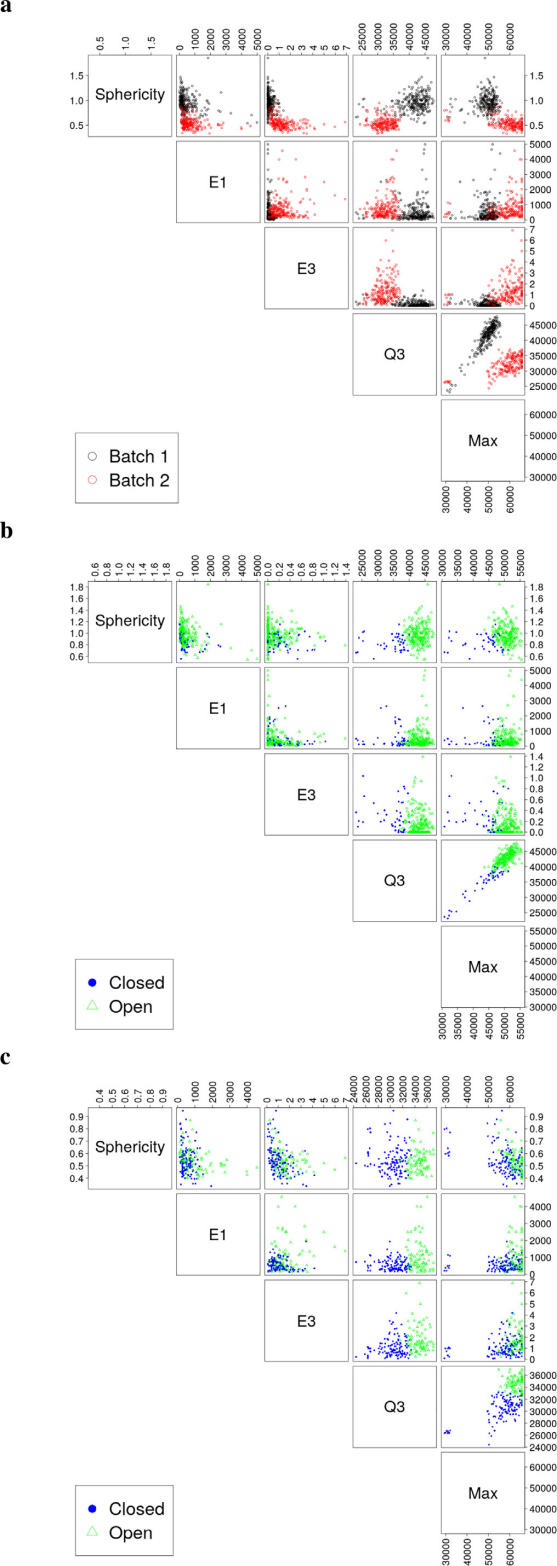
EPICS is able to account for batch effects in their original space. **(a)** Scatterplot matrix showing the features from Fig. 2, but with colorizations corresponding to the first and second experiments. The black and red open circles correspond to the first and second experiment, respectively. **(b, c)** Scatterplot matrices showing the chromatin domains from the first experiment **(b)** and from the second experiment **(c)**.

Our analysis shows that strong batch effects exist for 3D-EMISH data. The first 3D-EMISH experiment published in Trazaskoma *et. al.* [13] was 7×7×30 nm for each voxel, while the second experiment was 5×5×30 nm. After correcting for the differences in voxel resolutions during the reconstruction step, we observed that chromatin domains identified from the two batches can be clearly separated in the feature scatterplot matrix (Fig. 4a), indicating strong evidence of a batch effect. We confirmed this by building a LR model to predict the batch using the six variables identified by LASSO. This model was able to achieve about 98% and 99% overall accuracy on the training and validation data, respectively. Note that this accuracy was based off of the labels generated from k-means clustering. Thus, future analyses should account for different voxel resolutions.

To account for the batch effect, we used EPICS to first cluster per each batch using batch removal and *k*-means independently before combining the data for the LASSO. Batch removal was performed by subtracting the sample mean and standard deviation of for each respective feature by batch (Eq. 7). Typical batch removal requires the analysis to remain in an abstract space. However, we found that EPICS is still able to classify the open and closed chromatin domains in the original feature space (Figs. 4b & 4c). For instance, the blue solid dots are left of the green open triangles across batches for Q3 against Sphericity, E1, or E3. While the two experiments have different ranges for the features selected by LASSO (Figs. 4b & 4c), EPICS is able to use the clusters found from the independent batch removal step in the LR model using the original physical space for the variables (Fig. 2, Fig. 3b). Thus, EPICS is still able to capture the important biological features across batches in their original physical space and identify open or closed chromatin domains.

### 3.3 EPICS identifies chromatin domains from 3D-SIM data

**Fig. 5:**
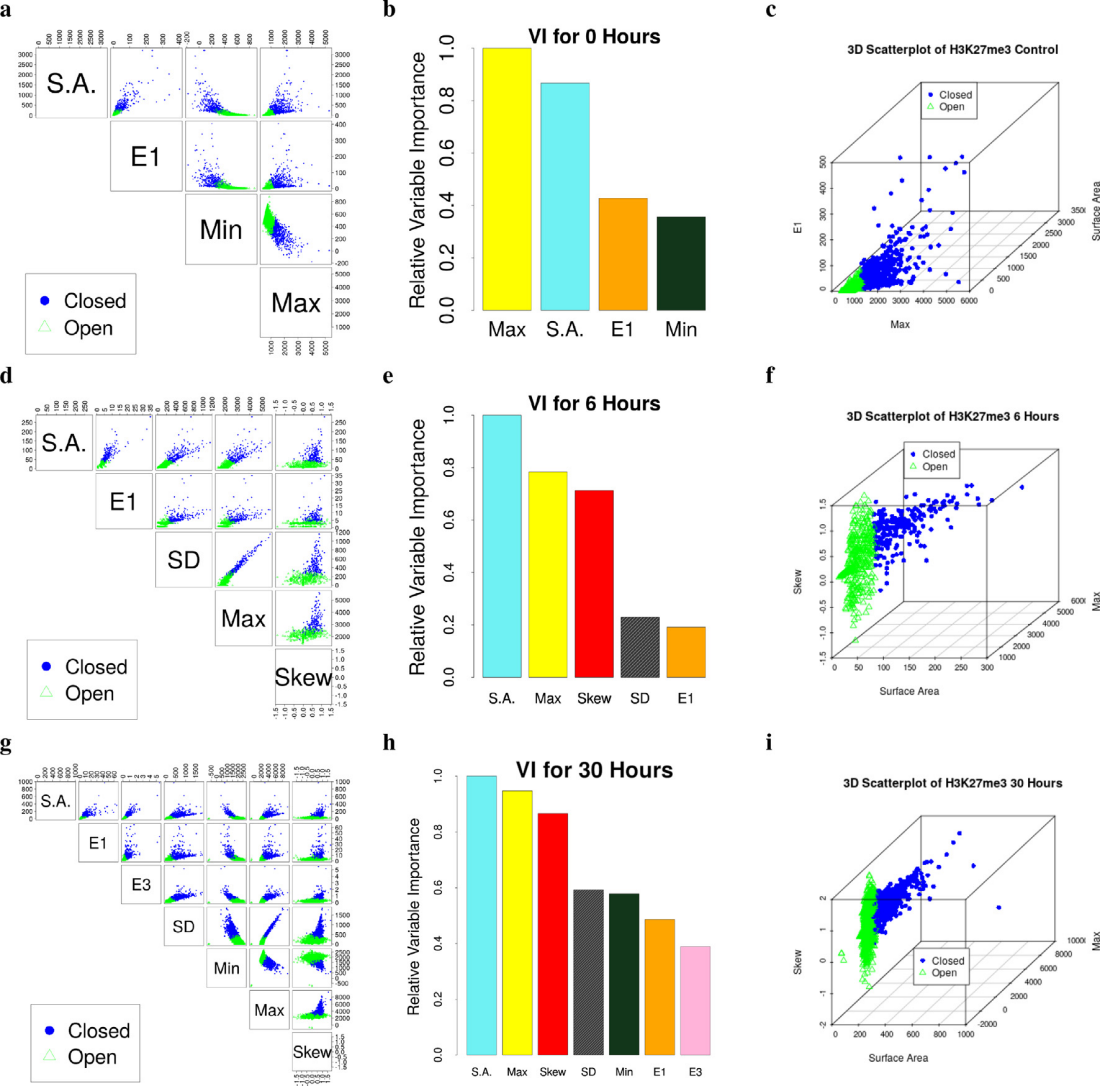
EPICS identified open and closed chromatin domains (CDs) from 3D-SIM data for H3K27me3 and a human colon cancer line across different treatments. Open and closed CDs are presented using green open triangles and blue closed circles, respectively. S.A., Surface Area. **(a, d, g)** Scatterplot showing the EPICS-identified important variables. **(b, e, h)** The relative variable importance (VI). **(c, f, i)** A 3D scatterplot of the top 3 most important variables clearly separating open and closed CDs for a given sample.

To test the general usability of EPICS, we applied EPICS to 3D image data generated from other techniques. Specifically, we applied EPICS to 3D-SIM generated immunostained images for repressive histone modification H3K27me3 in human colon cancer cell lines [25]. We identified open and closed chromatin domains of the cell under three different conditions: control, treated for 6 hours in auxin, and treated for 30 hours in auxin (Fig. 5, Supplementary Tables C6–C8). Each dataset was an image stack. Each voxel intensity value corresponds to a biological object as summarized in Fig. 1h. Large intensity values indicate more chromatin, while smaller values correspond to less. We processed the 3D-SIM data with the similar procedure for 3D-EMISH, as shown in parallel in Fig. 1. For example, we identified the important variables and characterized the physical properties of the H3K27me3-marked chromatin in these cancer cells under different treatments (Supplementary Tables C6–C8), and provided overall accuracy measures and confidence intervals (CIs) for each model’s performance (Supplementary Tables C9–C15). The smallest accuracy on the validation data was about 0.990 with an associated 95% CI of (0.980, 0.995). Note that the accuracy of these models was evaluated by using the clusters created by k-means. The learned labels were verified to be consistent through bootstrap sampling and obtaining similar cluster labels across the experiments (Tables C16 and C17 and Figs. B19–B21). Furthermore, the coefficient values are largely consistent across the bootstrapped samples (Tables C18–C20). Deeper investigations on the 6 hour model indicate that a more stable solution is viable using only the top 3 variables found by the LASSO (Table C21). Thus, one’s point of view of computational algorithms changes how one would utilize EPICS. If one wants an all-inclusive approach that requires no human inputs, EPICS provides results that are computationally valid and useful. However, if one wants to adhere to orthodox tenants (i.e. statistical significance), then the user is also able to investigate and fine-tune the final model produced by EPICS.

There are two primary biological insights obtained from these results. The first is the nature of open and closed chromatin domains at a coarser resolution using 3D-SIM relative to the finer resolution of 3D-EMISH. When compared to open chromatin domains, closed domains tend to have more chromatin material, a larger surface area, and have very dense sections within the domain. Thus, closed domains appear as large, dense, asymmetric chromatin. When compared to closed chromatin domains, open domains tend to have less chromatin material, less surface area, and a smaller major axis. Thus, open domains tend to be small, sparse, symmetric chromatin. The second biological insight is the increase in complexity required to classify open and closed chromatin domains across treatment types. There is evidence that chromatin domains exist across different treatments [25]. However, our models indicate that the chromatin domains are not static and remain unchanged across treatments. In fact, our model shows that the treatments might change the physical characteristics of the chromatin. In particular, since the 3D-SIM data has three treatments, we can quantify the differences between models for classifying the open and closed chromatin domains. For example, each of the 3D-SIM models retained surface area and the max intensity. However, the parameter values in our models are drastically different from one another. Such differences might be due to technical variances on the data or the model, or could be real biological results. Further investigation is needed on the EPICS results from 3D-SIM to provide insights to the biological nature of chromatin. In summary, these models reflect biological differences between the treatments. However, experimental validation in a wet lab is still required.

## 4 Discussion

In this work, we present a computational method, EPICS, which we developed to identify chromatin domains from 3D image data and to determine if identified chromatin domains are open or closed based on its physical representation from the images. We used data generated from 3D-EMISH and 3D-SIM techniques as two case studies to exemplify the workflow, functions, and results of EPICS, as well as the ability to correct for batch effects for 3D-EMISH data. More importantly, we demonstrated the ability of EPICS to explain and interpret the model and results. This allows researchers to learn the physical characteristics from super-resolution imaging data to understand the morphological properties of higher-order chromatin structures such as open and closed chromatin domains or active and inactive compartment structures. From the 3D-SIM data in cells under different treatments, we provided evidence that open and closed chromatin domains’ physical characteristics might dynamically change in time under treatments. Furthermore, we incorporated the tenants of explainability and interpretability into the development of EPICS to ensure the ML methods used are within the confines of XAI. To our knowledge, EPICS is the first and so far the only XAI method for analyzing 3D chromatin image data.

Specifically, we showed that EPICS is able to characterize open and closed chromatin domains from 3D-EMISH images at resolutions as small as 5×5×30 nm and 3D-SIM images at 39.5×39.5×125 nm. We identified the variables that were the most important to discriminate open and closed chromatin domains for each of our models and data sources. The LR model was able to achieve an overall classification rate of about 94% on the 3D-EMISH validation data, which were not used to build the model. The 3D-SIM model was able to achieve accuracies of at least 98% on the validation data. These accuracy rates were based upon using the *k*-means clustering labels. Thus, the models should generalize to outside data well. Therefore, we provide a computational framework that is able to describe and classify open and closed chromatin domains from these two different technologies.

Logistic regression (LR) is highly explainable since we can describe the exact relationship modeled and how the parameters of the model work. LR is interpretable since each parameter corresponds to a physical change in the probability of belonging to a particular class, and the final LR parameter values are shown in Table 1. Thus, LR is a prime example of a model which is able to satisfy the conditions of an XAI model.

EPICS handles the batch correction by clustering the data by batch before the model is built. This is vital as these chromatin domains were collected over two different datasets, but EPICS is able to classify the clusters using the LASSO and LR in their original unchanged space. The final LR model enables future analysts the ability to predict new observations without needing to do any batch correction for 3D-EMISH data.

Lastly, EPICS reduces model complexity by identifying the important features from the image data to consider as exemplified in Fig. B6. By removing less important variables, biologists are able to make more insightful and meaningful inferences. By using the LASSO, we are able to identify the relevant features for understanding the underlying biological properties of the chromatin domains. Thus, EPICS allows analysts to interpret the results in the original and physical units of the collected features.

This is the first work to characterize higher-order chromatin structures from image data in this manner. Chromatin structures from 3D-SIM data across different treatment for cells using volume were found to have statistically significant differences between the treatments [25]. Others used sphericity, surface area, and volume to find statistically significant differences between chromatin structures that have different number of domains [13]. However, statistical significance may not be sufficient to accurately classify different groups from one another. Further, none of these approaches use a large number of features to describe the physical characteristics of chromatin structures, nor do they use these features to identify clusters of open and closed chromatin domains. Thus, EPICS is the first approach to identify open and closed domains using the physical characteristics of chromatin.

The identification of open and closed chromatin domains from 3D chromatin image data using EPICS is analogous to the identification of A/B compartments from Hi-C data. We speculate that they refer to comparable structural information of chromatin states. However, due to lack of orthogonal information such as genomic coordinates for validation, the ground truth of what open/closed chromatin domains actually mean remains unclear. Further studies are needed for finding more biologically meaningful interpretation of these computationally-determined chromatin states.

There are three primary pitfalls of EPICS. The first is that some steps in the image pre-processing differ between the two technologies. This is primarily due to the nature of the different types of raw data. However, further improving the similarities in the algorithm would help ensure that EPICS treats the different raw data from different technologies as equitably as possible. The second is that not all of the variables found using the LASSO in EPICS are guaranteed to be statistically significant. Future work for identifying consistently statistically significant variables is an area of research. To that end, future versions of EPICS could reduce the number of variables the top 3. The third is the lack of genomic coordinate information in the two types of image data presented in this work. 3D-EMISH and 3D-SIM do not provide the genomic coordinates. Thus, we cannot directly compare Hi-C to our method. Other methods utilize probes that capture information across a small number of bases. These probes are then combined to obtain an entire trace. These traces can then be compared against technologies like Hi-C or ATAC-seq since they are approximately the same in terms of genetic resolution. These two families of technologies are usually compared visually or with a single metric like correlation (using their respective distance matrices). 3D-EMISH and 3D-SIM’s lack of genomic coordinates limits the possibility to further interpret the chromatin domain classification result of EPICS for functional association with the genome, and presents us from using orthogonal information such as Hi-C data to validate our image-based domain inference. However, if 3D-EMISH and 3D-SIM are developed to the point where we are able to identify probes at a higher resolution. Potential application of EPICS to other FISH-based spatial genomics data that barcode genomic information, such as seqFISH, ORCA, or MINA, can further improve the interpretability of EPICS for functional genomics studies. Nevertheless, considering that these pitfalls can be managed and improved in future work, EPICS potentially has a broad application in image-based spatial genomics data analysis.

## 5 Conclusion

EPICS allows us to understand the biological underpinnings of cells at a new level for investigation. Using our model for classifying chromatin domains from 3D-EMISH or 3D-SIM data would allow for deep understandings of a variety of chromatin structures. Unlike Hi-C, 3D-EMISH and 3D-SIM are able to provide physical representations of the chromatin. Further, 3D-EMISH and 3D-SIM provide a more direct measurement of chromatin than Hi-C. Thus, EPICS has the potential to yield new and meaningful biological insights for chromatin structures captured using 3D image data.

## CRediT authorship contribution statement

**William Franz Lamberti:** Conceptualization, Methodology, Software, Validation, Formal analysis, Investigation, Data curation, Visualization, Writing – original draft. **Chongzhi Zang:** Conceptualization, Writing – review & editing, Supervision, Project administration, Funding acquisition.

## Declaration of Competing Interest

The authors declare that they have no known competing financial interests or personal relationships that could have appeared to influence the work reported in this paper.
