# Supplementary material for: Extracting physical characteristics of higher-order chromatin structures from 3D image data
Source: Comput Struct Biotechnol J. 2022 Jun 20;20:3387–98. doi: 10.1016/j.csbj.2022.06.018 (PMC9260447; doi:10.1016/j.csbj.2022.06.018)
Supplement: Supplementary file 1 [file mmc1.pdf]

# Supplementary Data

## A Supplementary Text

### A.1 Bootstrap Experiment for Determining Clustering Labeling Consistency

To ensure that the clusters found during EPICS were consistent and not due to random chance, we performed the following procedure 100 times:

- Sample with replacement from the sample data $M$ times
- Cluster via $k-$means using sample of size $M$ from previous step
- Collect Jaccard's index (JI), accuracy, and balanced accuracy

For 3D-EMISH and 3D-SIM, $M$ =250 and $M$ =2500, respectively. After this process was repeated 100 times, the mean value was computed for JI, accuracy, and balanced accuracy. These results are summarized for 3D-EMISH and 3D-SIM in Tables C1 and C16, respectively.

### A.2 Bootstrap Experiment for Determining 3D-SIM Coefficient Consistency

To ensure that the coefficient estimated during EPICS were consistent, we performed the following procedure 100 times for each treatment:

- Sample with replacement from the sample data 2500 times
- Estimate the logistic regression (LR) model's coefficients estimated from the LASSO from the training data

We then calculated the mean and 2.5% and 97.5% quantiles summary statistics of the estimated from the 100 experiments. This is summarized in Tables C18 - C20. We also repeated the LR model for 6 hours using only the top 3 variables and validated the coefficient values (Table C21).

### A.3 Hi-C Compartment Assignment

We include a summary of the computation involved for Hi-C compartment assignment from Lieberman-Aiden *et. al.*[7]. We provided their framework here explicitly to precisely compare and contrast our approach to theirs. Their first step is to

$$\delta = \mathbb{C}_f - \Delta \tag{A1}$$

$$|\mathbb{C}| = \mathbb{C} - \delta \tag{A2}$$

$$\mathbb{X}_s = \mathcal{D}_E|\mathbb{C}| \tag{A3}$$

where $\Delta$ a $3 \times i$ is the location of each Fiducial bead in each tensor, $\delta$ is the correction factor to be used for each image after MERFISH is applied, $|\mathbb{C}|$ is the corrected distances, $\mathcal{D}_E$ calculates the Euclidean distance between the compartments, and $\mathbb{X}_s$ is the resulting $i \times i$ spatial distance matrix. They then estimate coefficients, $\hat{\beta}$, to obtain a normalized genetic distance matrix, $|\mathbb{X}_s|$, by performing:

$$\mathbb{X}_s = \vec{\beta}\mathbb{X}_g + \epsilon. \tag{A4}$$

$$\hat{\mathbb{X}}_s = \hat{\beta}\mathbb{X}_g \tag{A5}$$

$$|\mathbb{X}_s| = \frac{\mathbb{X}_s}{\hat{\mathbb{X}}_s} \tag{A6}$$

where $\mathbb{X}_g$ be the genetic distance $i \times i$ matrix. A genetic distance between two genes measure how similar a pair of genes and $\hat{\mathbb{X}}_s$ is the predicted $i \times i$ matrix. Since the Normalized matrix, $|\mathbb{X}_s|$, is not guaranteed to be 1, we need to perform these operations to guarantee that this occurs. Thus, they perform

$$|\mathbf{X}_s|_{i,i} = 1, \forall i = j. \tag{A7}$$

$$\mathbb{V} = \mathcal{V}|\mathbf{X}_s|, \tag{A8}$$

$$\rho_{i,j} = \frac{\mathbb{V}_{i,j}}{\mathbb{V}_{i,i} \times \mathbb{V}_{j,j}}, \forall i, j \tag{A9}$$

$$\vec{\lambda} = \mathfrak{P}_1 \rho \tag{A10}$$

$\mathcal{V}$ extracts the covariance matrix and $\mathfrak{P}_1$ performs principal component analysis and extracts only the first dimension's $i$ coefficient values and outputs them to the vector $\vec{\lambda}$. Lieberman-Aiden *et. al.*observed that the first eigenvector corresponds to compartment assignment[7]. Thus, they suggest that:

$$f(\lambda_i) = \begin{cases} A & \lambda_i > 0 \\ B & \lambda_i < 0. \end{cases} \tag{A11}$$

However, they make their most important inferences using a correlation matrix. Thus, they weaken their inferential capabilities by relying upon correlation[55, 56].

### A.4 Simulating Chromatins Data Exploration

This paper describe the first analysis to use shape metrics to classify CDs as open and closed. To this end, we simulated genomic structures using multivariate normal or Gaussian distributions to ensure that using shape metrics would be reasonable. The two multivariate normal distributions that were selected that shared the same mean, $\mu$. The covariance matrices of the first and second distribution were, respectively, $\Sigma_1 = \begin{bmatrix} 1 & 0 \\ 0 & 1 \end{bmatrix}$, and $\Sigma_2 = \begin{bmatrix} 0.001 & 0 \\ 0 & 0.001 \end{bmatrix}$. The first covariance matrix corresponds to open CDs, while the second corresponds to closed CDs. For each simulated

structure, we randomly selected 1000 observations. Using image operator notation to represent an image processing algorithm[35], these two distributions are then converted into 2D images using

$$\mathbf{b}[\vec{x}] = \Gamma_{>0}\mathbb{H}_{2,2}X, \tag{A12}$$

where $X$ is the input data, $\mathbb{H}_{2,2}$ converts the data into a 2D histogram and $\Gamma_{>0}$ is the threshold image operator. This equation converts numeric data of the sparse and dense simulated structures into image like those provided in Figures B1a and B1b, respectively. This is then repeated 100 times to collect 100 images of these simulated structures for a total of 200 images. From each of these images, a variety of shape metrics are collected such as area. A sample of these shape metrics are provided in Figure B1c with the red dots representing the sparse structure and the black dots representing the dense structure. Since the two classes of structures are separable, we could easily create a model to classify these simulated genomic structures.

## A.5 Metric Collection

### A.5.1 Shape Metrics

We collected the encircled image-histograms (EIs) using the algorithm described by Lamberti [36]. This algorithm results in a vector $\vec{c}_{EI}$ which contains the white and black pixel counts from image $\mathbf{u}[\vec{x}]$. These counts are the first two metrics, $\vec{m}_1$ and $\vec{m}_2$, respectively. The shape proportion (SP) value for a given image, $i$, is merely

$$\vec{m}_{3,i} = \frac{\vec{m}_{1,i}}{\vec{m}_{1,i} + \vec{m}_{2,i}}. \tag{A13}$$

The SP value is essentially the proportion of white pixels after applying the SPEI algorithm [36]. This SPEI algorithm puts a shape in its minimum encompassing circle. Then the circle is placed in its minimum encompassing square. However, since our images are in 3D, we extended the SPEI algorithm in the manner suggested by Lamberti. He suggests to use a sphere in place of the circle and a cube in place of the square. The EIs are the white and black pixel counts after applying SPEI [36]. Lamberti states that the metrics from SPEI are highly explainable and interpretable[29].

To obtain the eigenvalues, we performed

$$\vec{e}_i = \mathbb{E}_{(1,3)} V \mathbf{u}_i[\vec{x}], \tag{A14}$$

where $V$ collects the covariance matrix of the shape matrix and $\mathbb{E}_{(1,3)}$ calculates the first, second, and third eigenvalues of the resulting covariance matrix. The $j^{\text{th}}$ eigenvalue on image $i$ is $\vec{e}_{i,(j)}$.

The first, second, and third eigenvalues are metrics $\vec{m}_4$, $\vec{m}_5$, and $\vec{m}_6$, respectively. It is well-known that the eigenvalues of a covariance matrix correspond to the linear combination in the data which maximizes the variance for their respective dimension [57]. For instance, the first eigenvalue is the linear combination of the data which maximizes the first eigenvalue [57]. We also know that the linear projections, or eigenvalues, are orthogonal to one another [57]. Thus, the eigenvalues are measures of each of the three axes of our given shape. Lamberti states that the eigenvalues have a medium level of explainability and interpretability[29].

Sphericity, $\vec{m}_7$, was collected on image $i$ by

$$\vec{m}_{7,i} = \frac{\pi^{\frac{1}{3}} \times \left(6 \times \vec{m}_{1,i}\right)^{\frac{2}{3}}}{\vec{m}_{8,i}}, \tag{A15}$$

where $\vec{m}_{1,i}$ is the White EI or more commonly known as volume for 3D images and $\vec{m}_{8,i}$ is surface area. Values close to 1 are more spherical. Values farther away from 1 are less like spheres in shape. Sphericity is very similar in spirit to circularity. The clear definition make sphericity highly explainable. However, sphericity is only somewhat interpretable. For example, sphericity does not provide guidance for what to classify the shape of a CD with a value of 2. The CD is certainly less spherical than a CD with a value of 1. However, sphericity is not equipped to answer interpret the metric with any physical meaning beyond being more or less like a sphere.

Surface area, $\vec{m}_8$, was collected on image $i$ by

$$\vec{m}_{8.i} = \sum \triangleleft \mathbf{c}_i[\vec{x}] - \sum \mathbf{c}_i[\vec{x}] \tag{A16}$$

Surface are is both highly explainable and interpretable. It is explainable since we can accurately describe how to calculate it. It is interpretable since surface area describes the amount of space the

CD's outermost layer occupies.

### A.5.2 Intensity Metrics

The features for intensity were the mean, standard deviation (SD), median, first quantile (Q1), third quantile (Q3), maximum, minimum, skewness, kurtosis, percentage greater than 24,000, and percentage greater than 30,0000 of the object's pixel intensity values. The object's pixels were extracted by using the segmented shape. Explicitly, for a given image and mask, metrics $\vec{m}_{9,i}$ to $\vec{m}_{19,i}$

$$\vec{m}_{9,i} = \mathcal{M}_{\mathbf{u}_i[\vec{x}]}\mathbf{r}_i[\vec{x}], \tag{A17}$$

$$\vec{m}_{10,i} = \mathcal{T}_{\mathbf{u}_i[\vec{x}]}\mathbf{r}_i[\vec{x}], \tag{A18}$$

$$\vec{m}_{11,i} = \mathbb{M}_{\mathbf{u}_i[\vec{x}]}\mathbf{r}_i[\vec{x}], \tag{A19}$$

$$\vec{m}_{12,i} = \mathbb{Q}_{\mathbf{u}_i[\vec{x}],0.25}\mathbf{r}_i[\vec{x}], \tag{A20}$$

$$\vec{m}_{13,i} = \mathbb{Q}_{\mathbf{u}_i[\vec{x}],0.75}\mathbf{r}_i[\vec{x}], \tag{A21}$$

$$\vec{m}_{14,i} = \bigvee_{\mathbf{u}_i[\vec{x}]} \mathbf{r}_i[\vec{x}], \tag{A22}$$

$$\vec{m}_{15,i} = \bigwedge_{\mathbf{u}_i[\vec{x}]} \mathbf{r}_i[\vec{x}], \tag{A23}$$

$$\vec{m}_{16,i} = \frac{\Sigma(\mathbf{r}_i[\vec{x}] - \vec{m}_{9,i})^3}{(\Sigma\mathbf{u}_i[\vec{x}]) \times \vec{m}_{10,i}^3}, \tag{A24}$$

$$\vec{m}_{17,i} = \frac{\Sigma(\mathbf{r}_i[\vec{x}] - \vec{m}_{9,i})^4}{(\Sigma\mathbf{u}_i[\vec{x}]) \times \vec{m}_{10,i}^2} - 3, \tag{A25}$$

$$\vec{m}_{18,i} = \frac{\Sigma\Gamma_{>24000,\mathbf{u}_i[\vec{x}]}\mathbf{r}_i[\vec{x}]}{\Sigma\mathbf{u}_i[\vec{x}]}, \tag{A26}$$

$$\vec{m}_{19,i} = \frac{\Sigma\Gamma_{>30000,\mathbf{u}_i[\vec{x}]}\mathbf{r}_i[\vec{x}]}{\Sigma\mathbf{u}_i[\vec{x}]}. \tag{A27}$$

where $\mathcal{M}$, $\mathcal{T}$, $\mathbb{M}$, $\mathbb{Q}$, $\bigvee$, and $\bigwedge$ are the mean, standard deviation, median, quantile, max, and min operators, respectively. All of these operators are only extracted on the 1's of image $\mathbf{u}[\vec{x}]$, which ensures that only the colors of object are considered for a given input image. Note that a intensity value has different meanings across technologies. For example, small values for 3D-EMISH mean that more of the chromatin is present and larger values mean that there is more. Conversely, small values for 3D-SIM mean that there is less genetic material, while large values mean that there is more. This is summarized in Figure 1h The following paragraphs describe each metric in details. This will show how our intensity features have an interpretable and explainable meanings.

The mean value for a given channel indicates how much of that color is in the respective channel. For 3D-EMISH, large values indicate that target chromatin is not present in the image, while small correspond to the image having larger amounts of a given genetic target. The opposite is true for 3D-SIM. Larger values mean that there is more genetic material, and smaller values mean that there is less genetic material. Thus, the mean intensity value is explainable since it describes how it captures the typical amount of the genetic target. It is interpretable since the mean intensity value corresponds to physical definitions about the genetic target of interest. The median intensity value follows a similar logic to the mean.

The SD indicates how much the presence of a color changes throughout an image. A large SD indicates that the presence of the intensity of a color changes dramatically, while small a SD corresponds to a fairly uniform representation of the intensity of a color. Thus, the SD intensity value is explainable since it describes how it the amount of genetic target varies. It is interpretable since the SD intensity value corresponds to physical definitions regarding the variation of the

genetic target of interest in a given object.

The Q1 and Q3 values indicate the amount of genetic material that separates the sample observation's intensity distribution into the $25^{\text{th}}$ and $75^{\text{th}}$ quantiles, respectively. For 3D-EMISH, large intensity values indicate that target chromatin is not present in the image, while small correspond to the image having larger amounts of a given genetic target. For 3D-SIM, larger intensity values mean that there is more genetic material. Smaller values mean that there is less genetic material. Thus, the quantile values are explainable since it describes the dividing point of the sample distribution of an observation of the genetic target. It is interpretable since quantile values corresponds to physical definitions about the genetic target of interest.

The max and min values indicate the maximum and minimum intensity values observed in a given observation, respectively. For 3D-EMISH, large values indicate that target chromatin is not present in the image, while small correspond to the image having larger amounts of a given genetic target. For 3D-SIM, larger intensity values mean that there is more genetic material. Smaller values mean that there is less genetic material. Thus, the max and min are explainable since it describes the least and greatest amount of the genetic target in a given observation, respectively. It is interpretable since max and min values corresponds to physical definitions about the genetic target of interest.

The skewness values, $\vec{m}_{16}$, indicate how much the values of the target CD intensity values differ from a symmetric distribution (assuming that the target CD's distribution of intensity values is unimodal). A value of zero means that the CD's intensity values are fairly symmetrical, while non-zero values indicate that the distribution has a long tail. Negative values indicate that the left tail is longer, while positive values indicate that the right tail is longer. Thus, negative values indicate that there are outliers of dense areas of genetic material. Positive values indicate that there are outliers of sparse areas of genetic material. Therefore, skewness is explainable since

it describes an aspect of the CD's intensity distribution. It is interpretable since it indicates the amount of genetic material present.

The kurtosis values, $\vec{m}_{17}$, indicate the presence of outliers[58]. Assuming that the intensities follow a normal distribution, a value close to 0 indicates that there are no outliers. Non-zero values indicate the presence of outliers. In other words, a value close to 0 indicates that the CD is has a very consistent amount of genetic density. Non-zero values indicate the presence of sections of the CD that greatly deviate from the typical genetic density. Therefore, skewness is explainable since it describes an aspect of the CD's intensity distribution. It is interpretable since it indicates the presence of abnormal sections in the CD.

The last two metrics are merely the proportions of the intensity values greater than 24,000 or 30,000. These metrics were only used for 3D-EMISH. Large values indicate that the CD is sparser, while smaller values indicate that the CD is denser. Therefore, these proportions are explainable since it describes the amount of the CD that is sparse. They are interpretable since it indicates the proportion of the CD that is sparse.

### A.6 Extended Results for 3D-EMISH

The resulting LASSO plot for evaluating the choice in $\lambda$ for 3D-EMISH is provided in Figure B2. The bottom axis displays the log of the tuning parameter. The y-axis displays the misclassification error for varying tuning parameter values. The top axis displays the number of features retained in the model. The left dotted line displays the optimal $\lambda$ value. The right dotted line displays the $\lambda$ within 1 standard error of the optimal one. The red dot is the mean misclassifcation for a given $\lambda$. The error bars are the associated values obtained across the 10-folds. The optimal value was estimated to be about 0.01. This was used for all of the 3D-SIM experiments since it is a more

prudent choice of $\lambda$.

Table C1 provides the Jaccard index (JI), accuracy, and balanced accuracy for the bootstrap experiment across the replicates. Table C3 provides the numerical values for the relative VI. For example, E1 and E3 are only about 30% as important as the Q3 intensity.

Tables C4 and C5 provide the confusion tables for the training and validation data. Since we have large diagonal values with smaller non-diagonal values, we have evidence that the model performs well on the data used to build the model and data that the model was not trained on.

### A.7 Extended Results for 3D-SIM

One of the notable choices made for the 3D-SIM models was the $\lambda$ parameter for all of the models as seen in Figures B3, B4, and B5. These figures have similar interpretations as provided in Figure B2. While the procedure produced different final values (which provides additional, albeit weaker, evidence that the open and closed CD's physical characteristics are changing). However, across all of the data sets (including 3D-EMISH), the same general shape was provided. Further, the authors of the LASSO have stated that the more appropriate value for the LASSO is the larger value suggested value since it "errs on the side of parsimony"[40]. In other words, assuming that the evaluation metric is "reasonablly" close, choosing the simpler model is preferable since it favors a simpler model. To that end, we selected the most conservative $\lambda$ value across all of the experiments (about 0.01), as the value for the 3D-SIM models. This value favors a more conservative model that actively avoids over-fitting to the data, while also providing more interpretable and explainable models since less variables are needed to classify open and closed CDs. This choice of a more conservative value for  was also motivated by a personal effort to provide a more prudent solution in an effort to temper our model performance.

Tables C6-C8 provides the numerical values for the relative VI for the control, 6 hour treatment, and 30 hour treatment, respectively. Max and Surface Area remain the most important variables across the three, but the number of important variables increases with the length of the treatment.

Tables C9- C14 provide the confusion tables for the training and validation data across the control, 6 hour treatment, and 30 hour treatment cells. Since we have large diagonal values with smaller non-diagonal values for each confusion matrix, we have evidence that the model performs well on the data used to build the model and data that the model was not trained on.

Table C16 provides the Jaccard index (JI), accuracy, and balanced accuracy for the bootstrap experiment across the treatments.

### A.8 Image Processing for 3D-EMISH

The exact image processing steps differ from the more general framework provided in the main text in Equations 1 - 4. Specifically, we first apply

$$\mathbf{s}[\vec{x}] = \mathcal{S}_{0.1}(\mathbf{t}[\vec{x}] \otimes \mathbf{g}[\vec{x}]) \tag{A28}$$

where $\mathbf{t}[\vec{x}]$ is the input image that contain a chromatin structure (CS), $\otimes$ is the convolution operator, $\mathbf{g}[\vec{x}]$ is the local minimum operator of a $2 \times 2 \times 2$ voxel, $\mathcal{S}_{0.1}$ is the Gaussian smoothing operator with a standard deviation of 0.1, and $\mathbf{s}[\vec{x}]$ is the resulting smoothed image. We first removed spurious noises using the local minimum using a $2 \times 2 \times 2$ window. We then further smoothed the image using a Gaussian operator to remove the block-like structure created by the previous operation. Examples of the input image and resulting image are provided in Figures B7 and B8, respectively.

Next, we performed

$$\{\mathbf{o}[\vec{x}]\} = \Gamma_{\mathfrak{O}}\{\mathbf{s}[\vec{x}]\} \tag{A29}$$

where $\Gamma_{\mathfrak{O}}$ applies Otsu thresholding to each slice of the input smoothed image-stack. This operation extracts the structure from the background without the need for human inputs. An example of this result is provided in Figure B9.

Next, we applied

$$\mathbf{i}[\vec{x}] = \mathcal{I}_{(1)} \triangleright_1 \mathbf{o}[\vec{x}] \tag{A30}$$

where $\mathcal{I}_{(1)}$ extracts the largest object from the image-stack and $\triangleright_1$ erodes the isolated object 1 times. We first eroded the image isolate spurious aspects of the chromatin structure. We then isolated the largest object from the surrounding noise in the image. An example of this result is provided in Figure B10.

We then applied

$$\mathbf{m}[\vec{x}] = \mathbb{I}_B \mathbf{i}[\vec{x}] \tag{A31}$$

where $\mathbb{I}_B$ interpolates the object using the other slices to construct the missing slices. We performed this step to provide an estimate of the chromatin physical structure in physical space. An example of this result is provided in Figure B11.

To determine the number of CDs, we then need to apply the mask to the original image. We do this by

$$\mathbf{c}[\vec{x}] = \mathbf{t}[\vec{x}] \times \mathbf{i}[\vec{x}] \tag{A32}$$

$$\vec{c} = \mathfrak{K}_{K,10} \mathbb{P}_{0.60,1} \mathbf{c}[\vec{x}] \tag{A33}$$

where $\mathbb{P}_{0.60,1}$ finds the local max peaks of the structure from the input image while ignoring those intensities less than the $60^{th}$ percentile of intensity values of the structure while requiring a distance of 1 voxel for each peak. The next image operator, $\mathfrak{K}_{K,10}$ performs $k$-means clustering on the potential peaks to identify the centers of each domain with automatic cluster determination. We consider up to 10 potential clusters. This outputs $k$ centers in a vector $\vec{c}$. These operators were performed to find the center of each CD in the CFS. We then needed to extract the CDs in the correct units. Thus, we performed:

$$\{\mathbf{p}[\vec{x}]\} = \mathfrak{K}_{K,\vec{c}}\mathbb{P}_{0.60,1}\mathbf{c}[\vec{x}] \tag{A34}$$

$$\mathbf{d}[\vec{x}] = \mathbb{I}_{B}\mathbf{p}[\vec{x}] \tag{A35}$$

where we first predicted which domain each core of the structure in $\mathbf{i}[\vec{x}]$ belonged to in Equation A34. We then interpolated in Equation A35 as was done previously in Equation A31. An example of the discovered CDs is provided in Figure B12.

### A.9 Image Processing for 3D-SIM

There are multiple targets in the colon cancer cell image, this differs from the data obtained from 3D-EMISH data. The 3D-EMISH images had a single target in each image. Thus, some of the image processing steps need to be altered for us to collect the metrics.

Since the 3D-SIM images have three channels, we first need to relevant channels. To that end, we first apply

$$\mathbf{r}[\vec{x}] = \begin{Bmatrix} 1 \\ \varnothing \\ \varnothing \end{Bmatrix}\mathbf{t}[\vec{x}] \tag{A36}$$

$$\mathbf{b}[\vec{x}] = \begin{Bmatrix} \varnothing \\ \varnothing \\ 1 \end{Bmatrix} \mathbf{t}[\vec{x}] \tag{A37}$$

where we extracted the blue channel for the DAPI stained intensities and red channel for H3K27me3 intensities. $\mathbf{r}[\vec{x}]$ and $\mathbf{b}[\vec{x}]$ are shown together in Figure 1e. The DAPI channel will be used as a mask to help remove the spurious signals outside of the nucleus. We then applied Equation A28 to smooth $\mathbf{b}[\vec{x}]$ and obtain $\mathbf{s}[\vec{x}]$ as seen in Figure B13.

We then applied

$$\mathbf{n}[\vec{x}] = \mathcal{B} \triangleright_5 \mathcal{B}\mathcal{I}_{(1)}\Gamma_{Q_{0.80}>}\mathbf{s}[\vec{x}] \tag{A38}$$

where $Q$ is the quantile operator, $\mathcal{B}$ is the binary fill hole operator, and $\triangleright$ is the erosion operator in order to extract mask of nucleus to extract only relevant H3K27me3 signals. The quantile operator was applied to ensure that only the strongest signals were retrained from the original image. The isolation, binary fill holes, and erosion operators were applied to help identify the nucleus while also filling in the holes missed by the DAPI staining to ensure that every part of the nucleus is extracted. An example is shown in Figure B14.

We then need to extract the relevant H3K27me3 signals. To that end, we apply:

$$\mathbf{r'}[\vec{x}] = \mathbf{n}[\vec{x}] \times \Gamma_{>59}\mathbb{H}_{R,0,255}\mathbf{r}[\vec{x}] \tag{A39}$$

where $\mathbb{H}$ rescales the intensities from 0 to 255 in order to rescale and isolate the relevant signals. This results in an image of the mask of each CD in a single image as seen in Figure B15.

To extract the relevant image masks for the individual CDs, we then apply

$$\{\mathbf{s}_i[\vec{x}]\} = \mathcal{I}\mathbf{r'}[\vec{x}] \tag{A40}$$

which provides the set of individual CD masks such that $i \in \{1, 2, ..., n\}$. We then apply Equation A35 to $\mathbf{r}[\vec{x}] \times \{\mathbf{s}_i[\vec{x}]\}$ and $\{\mathbf{s}_i[\vec{x}]\}, \forall i$ to interpolate the image to ensure that each voxel is approximately the same in the $x - y - z$ directions. Figure B16 provides an example of all the CDs extracted from the example input image.

We then extract the shape and intensity metric using Equation 5. Lastly, we then apply Equations 7 - 15 as in the usual case to classify the open and closed objects. However, we only have one experiment or batch in this case. Conversely, we have three different treatments. Thus, we will build three separate models for each of the three cells: the control, the 6 hour treatment, and the 30 hour treatment.

# B Supplementary Figures

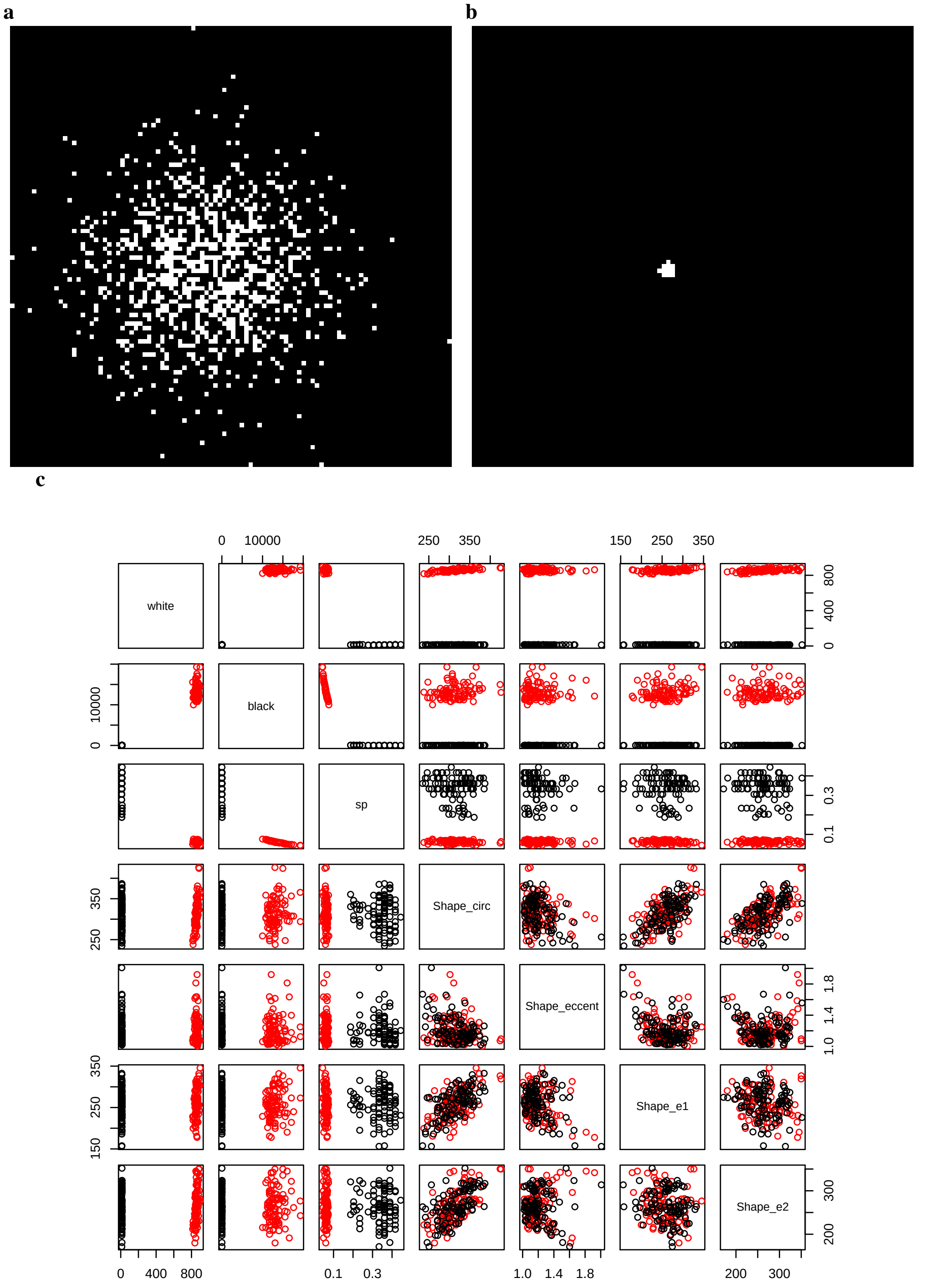


Figure B1: Figure shows examples of the dense and spare simulated genomic structures in Figures B1a and B1b. The resulting shape metrics from 200 simulated structures in Figure B1c shows that the two classes are separable. **(a)** A simulated sparser distribution. **(b)** A simulated dense distribution. **(c)** The collected shape metrics.

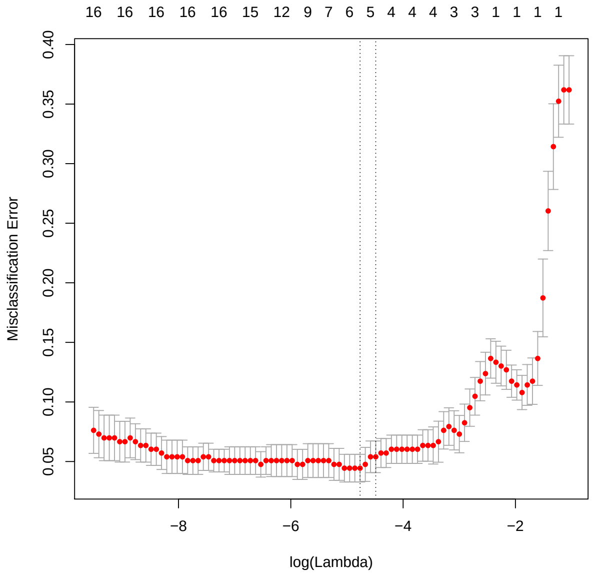


Figure B2: Figure shows the use of 10-folds CV to help select the tuning parameter for the 3D-EMISH data.

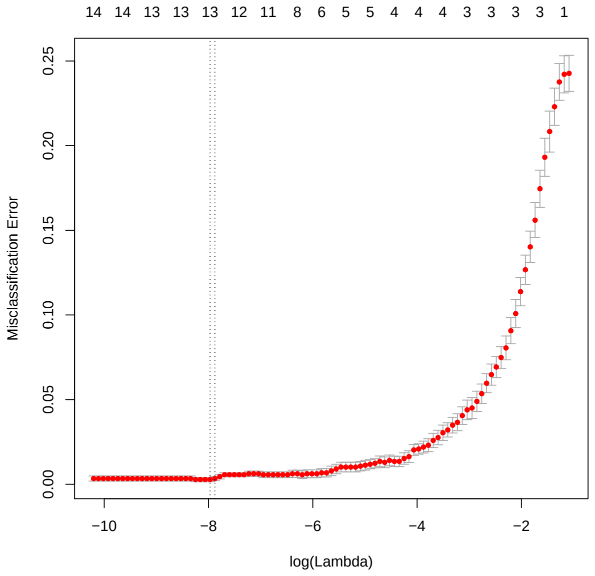


Figure B3: Figure shows the use of 10-folds CV to select the tuning parameter for the control 3D-SIM data.

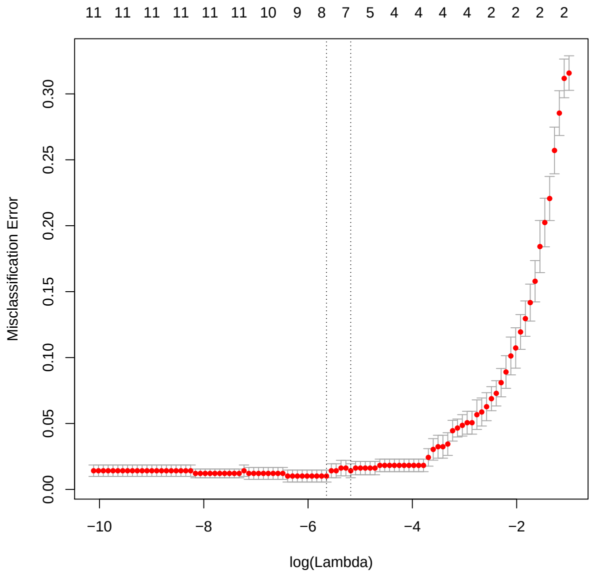


Figure B4: Figure shows the use of 10-folds CV to help select the tuning parameter for the 6-hour treatment 3D-SIM data.

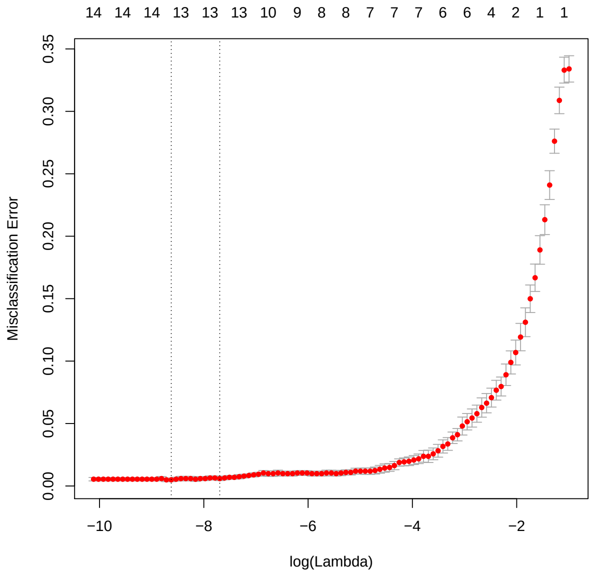


Figure B5: Figure shows the use of 10-folds CV to help select the tuning parameter for the 30-hour 3D-SIM data.

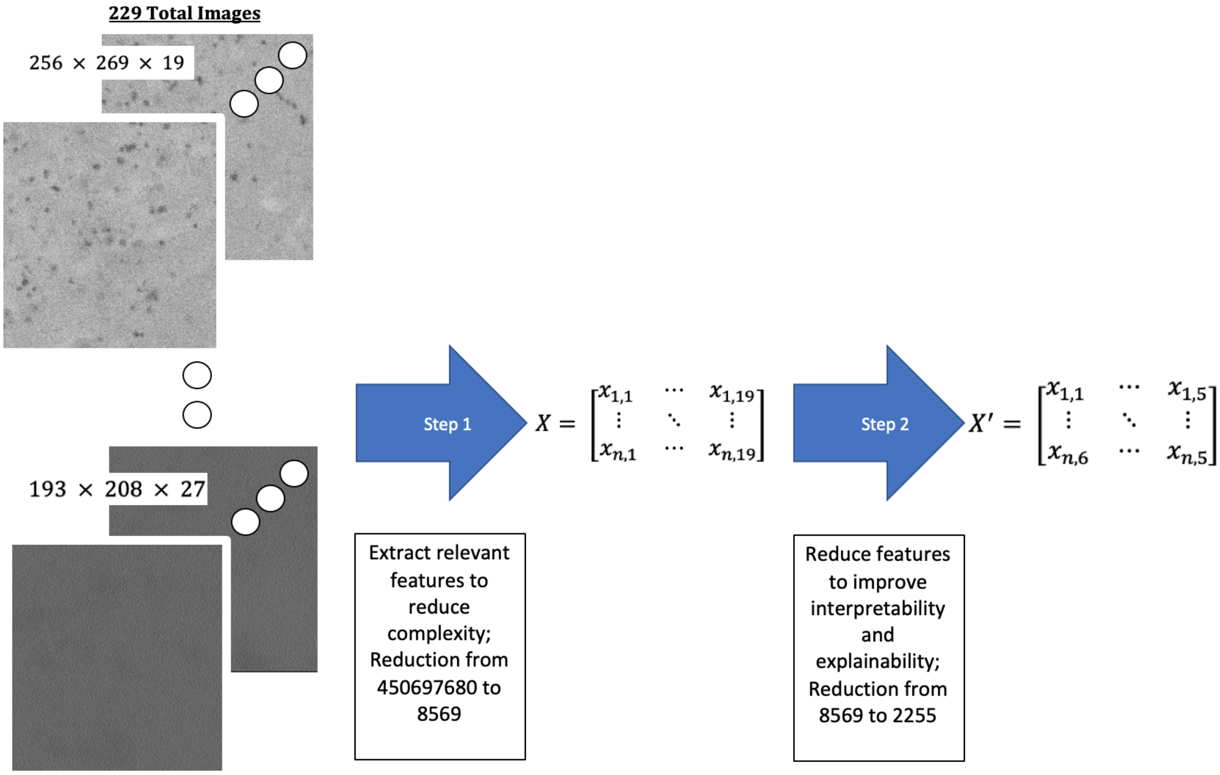


Figure B6: EPICS reduces complexity by removing less important variables by extracting shape and intensity image features and then using the LASSO ML algorithm as exemplified with the 3D-EMISH data.

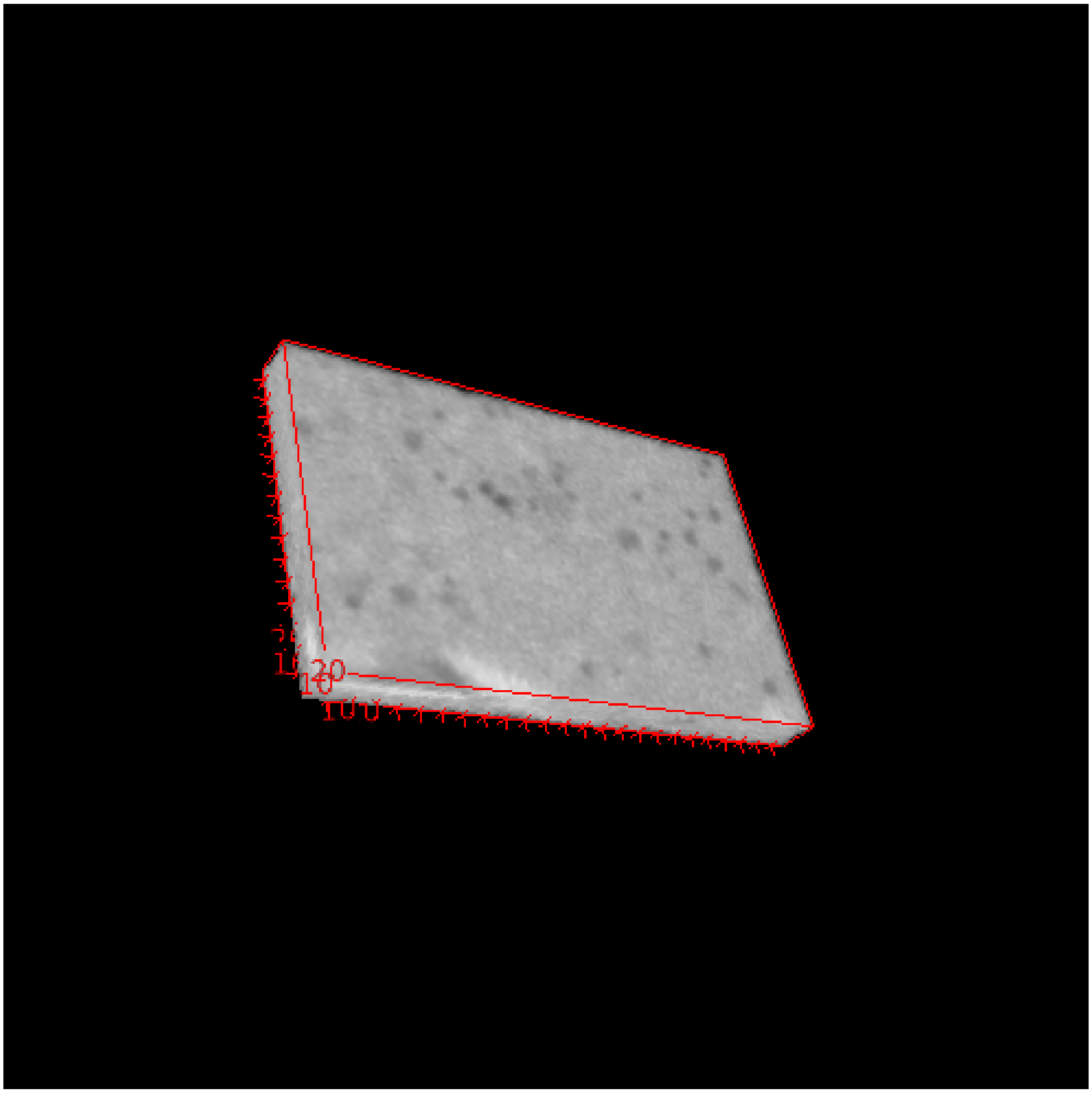

Figure B7: Example of input image for 3D-EMISH.

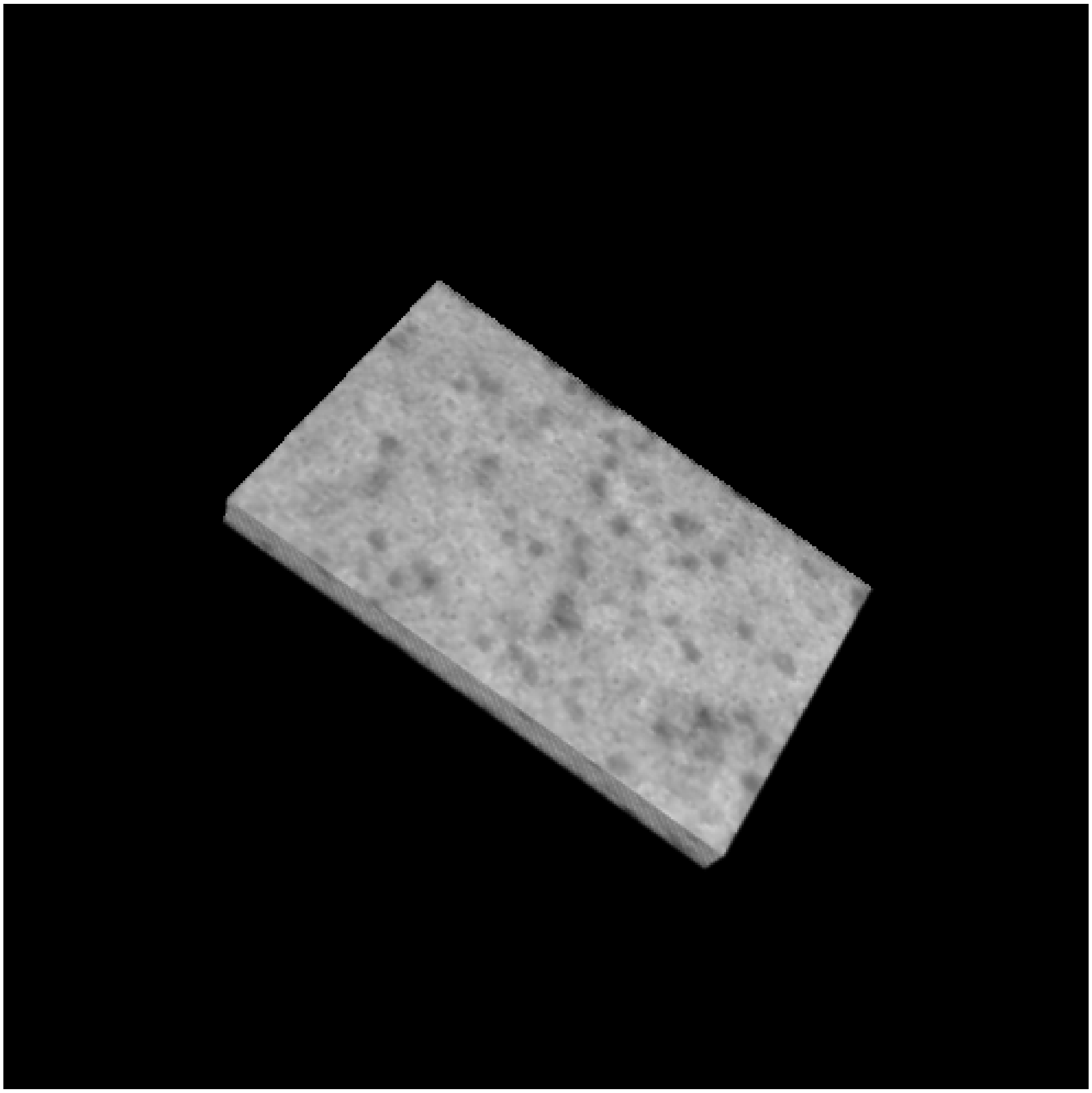
Figure B8: Example of smoothed 3D-EMISH image.

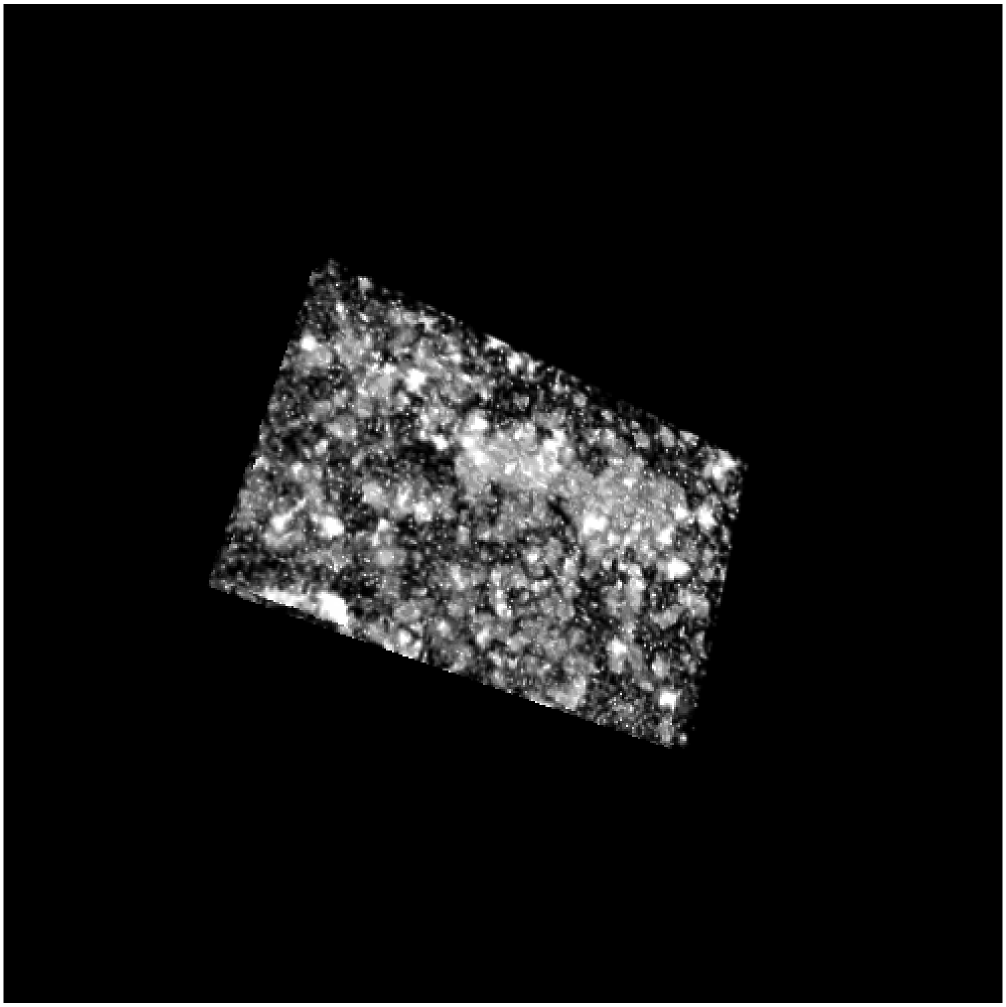

Figure B9: Example of isolated signals from 3D-EMISH example image.

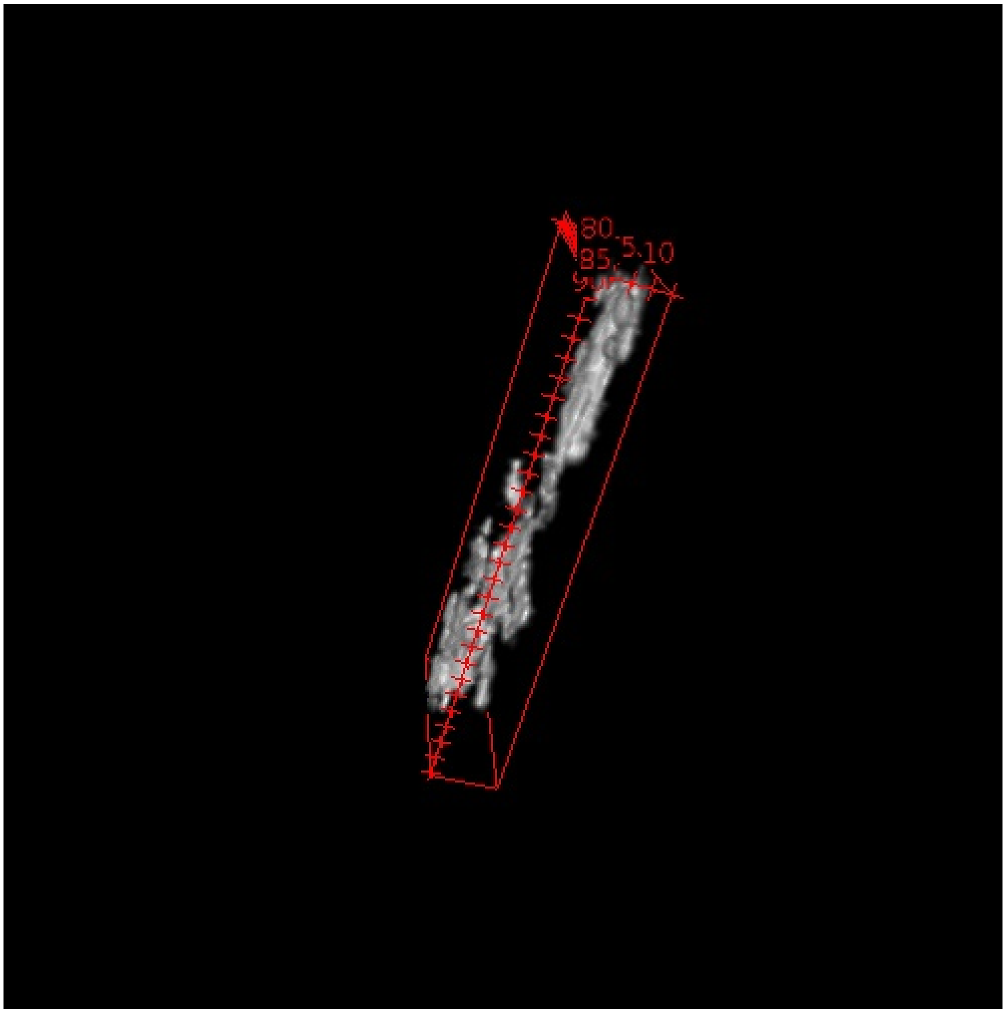


Figure B10: Example of largest isolated object from 3D-EMISH example image.

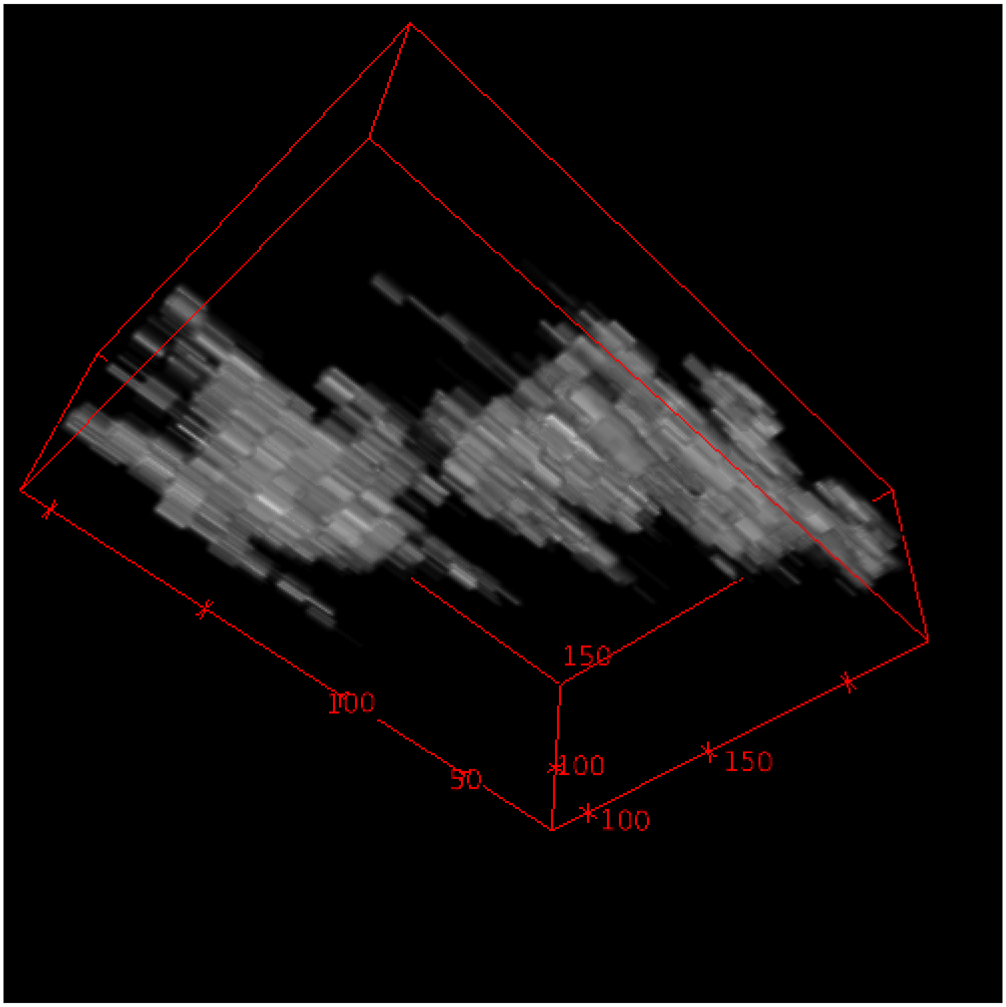


Figure B11: Example of reconstructed object of interest from 3D-EMISH data.

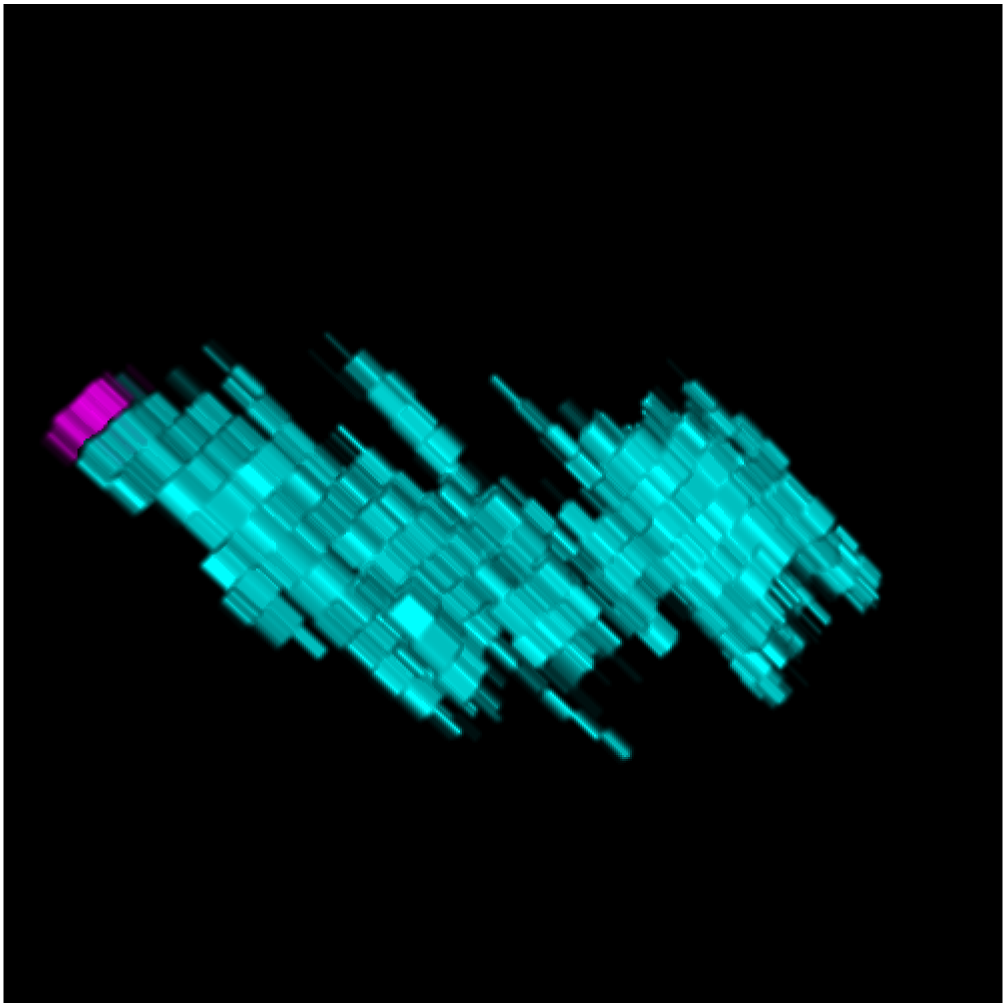

Figure B12: Example of CDs identified from example input 3D-EMISH data. Each different color represents a unique CD.

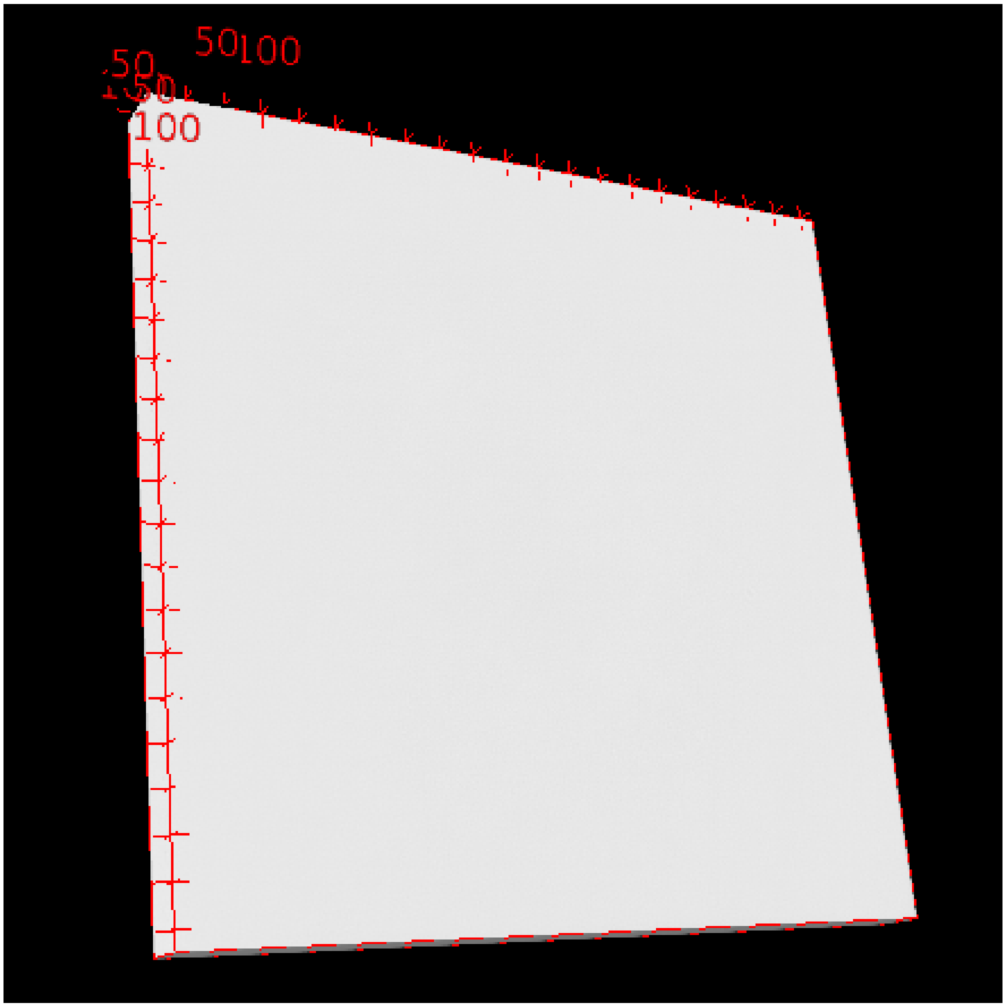


Figure B13: Smoothed image of the DAPI stained image from the 3D-SIM example. The input image was the example image from Figure 1e.

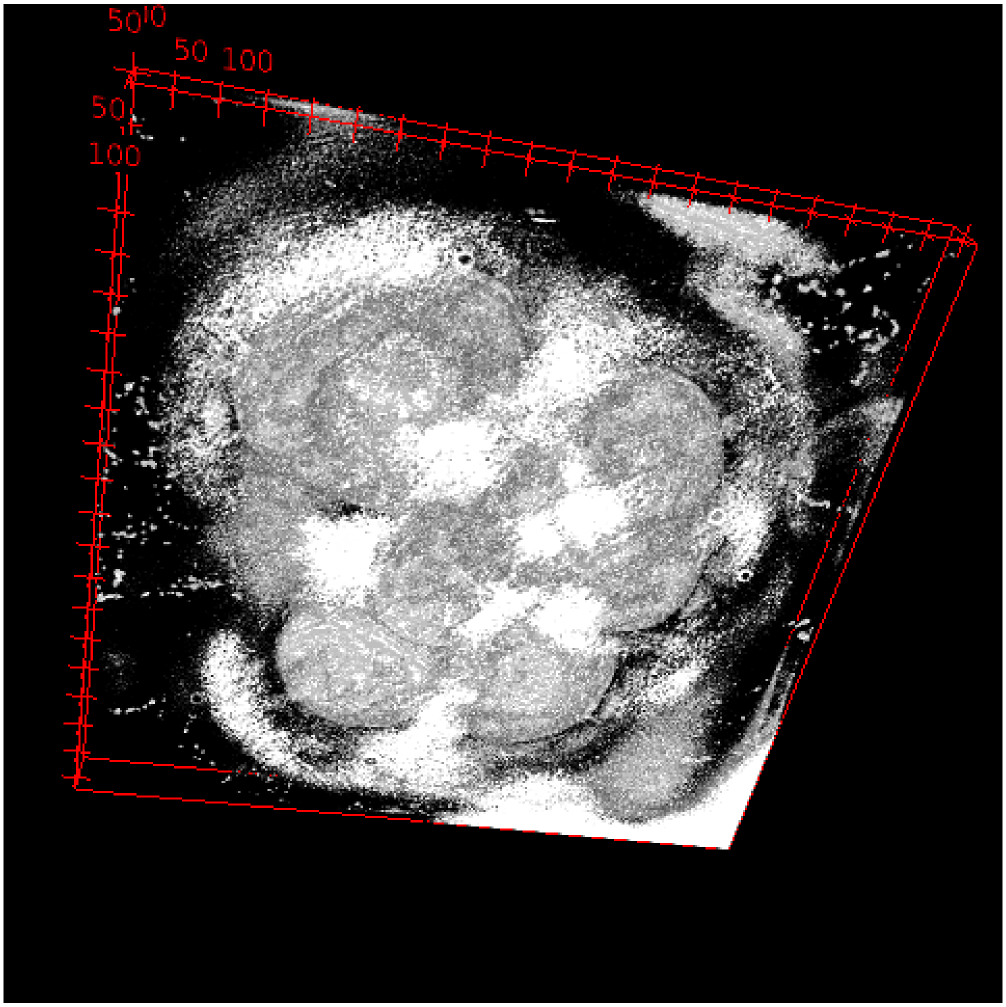


Figure B14: Example of identified mask from the DAPI stained image.

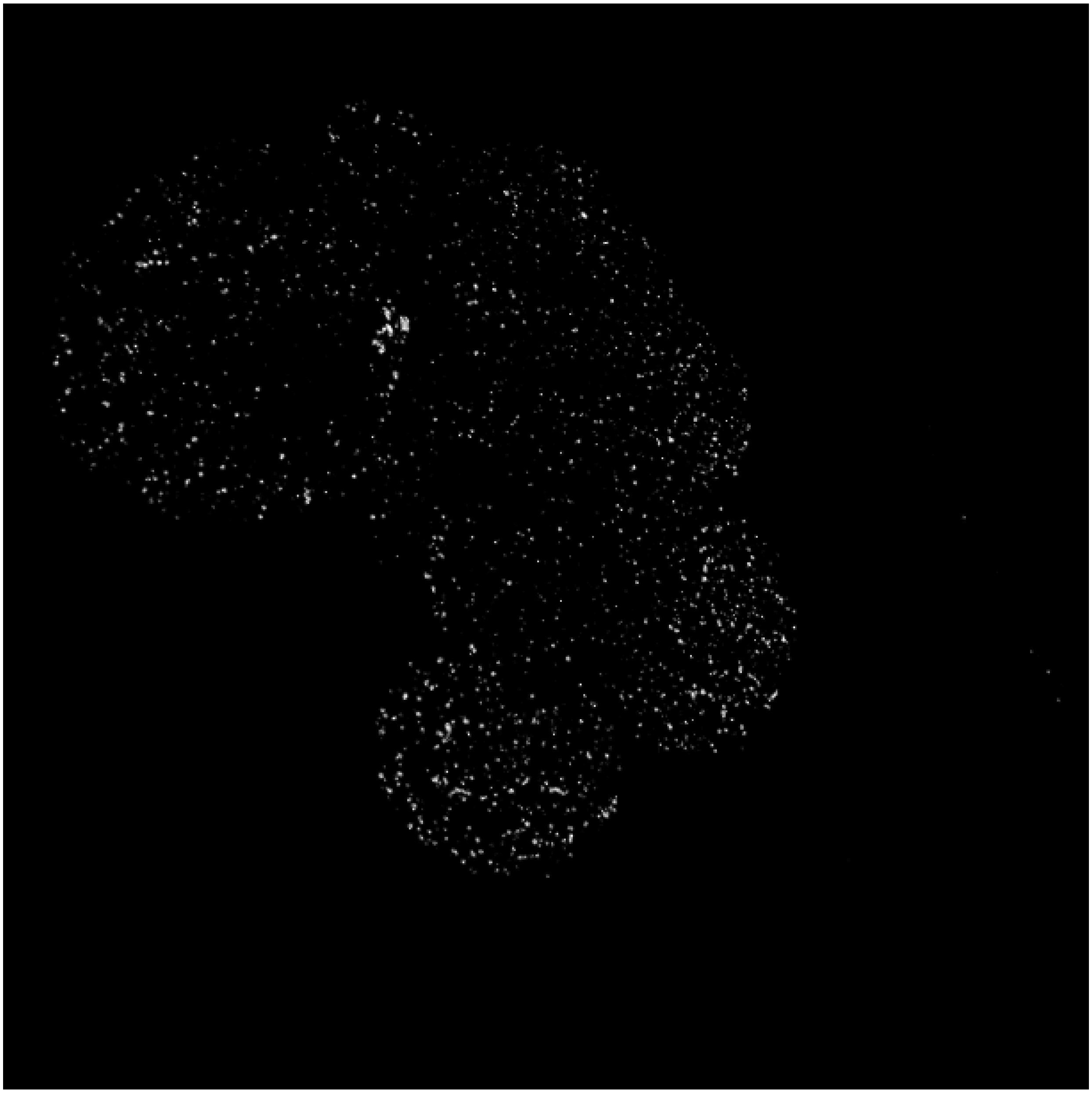

Figure B15: Example of the extracted signals of interest from the 3D-SIM image only within the nucleus.

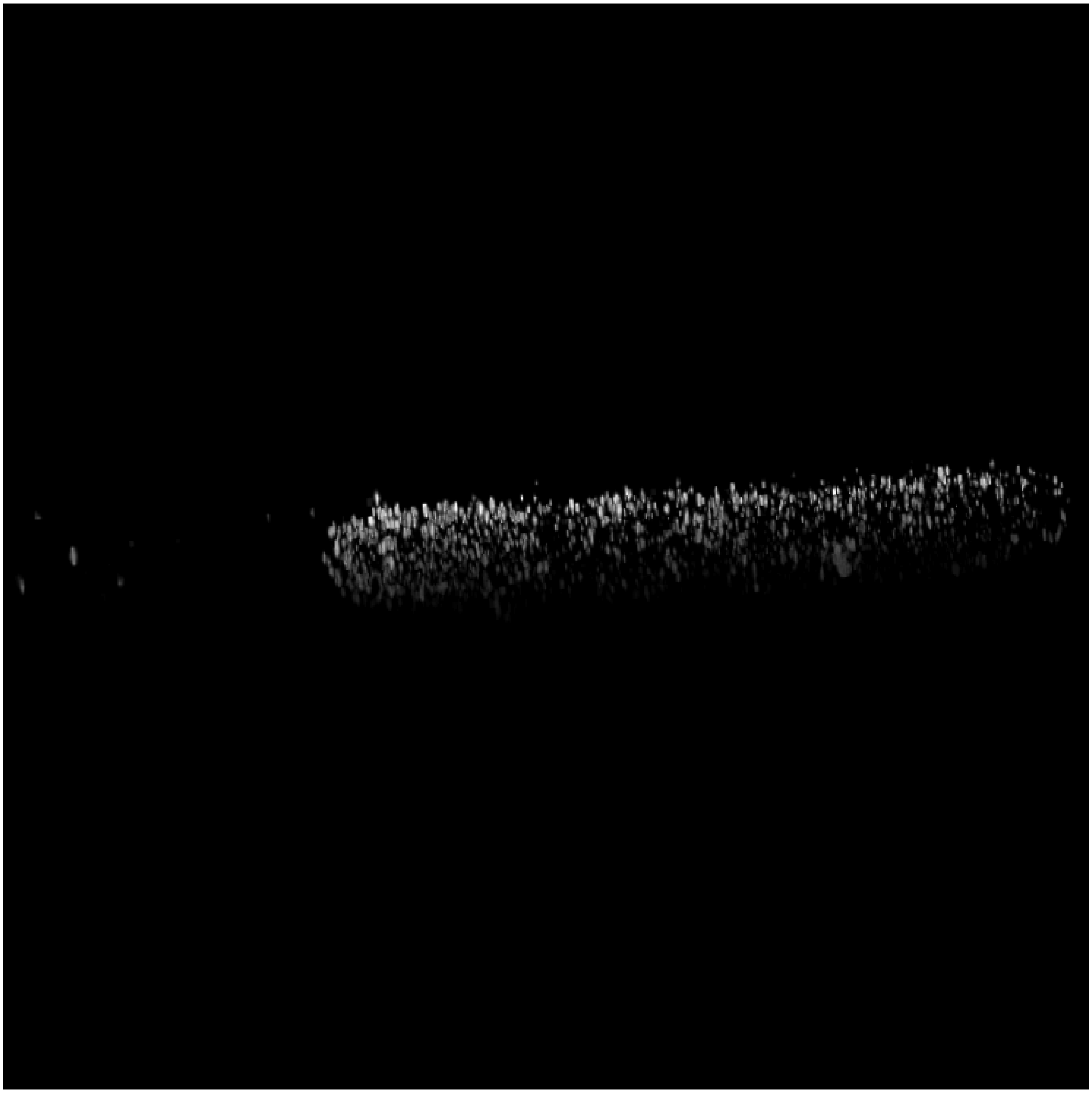

Figure B16: All of the CDs extracted from the example input 3D-SIM image. Each different grayscale intensity represents a unique CD.

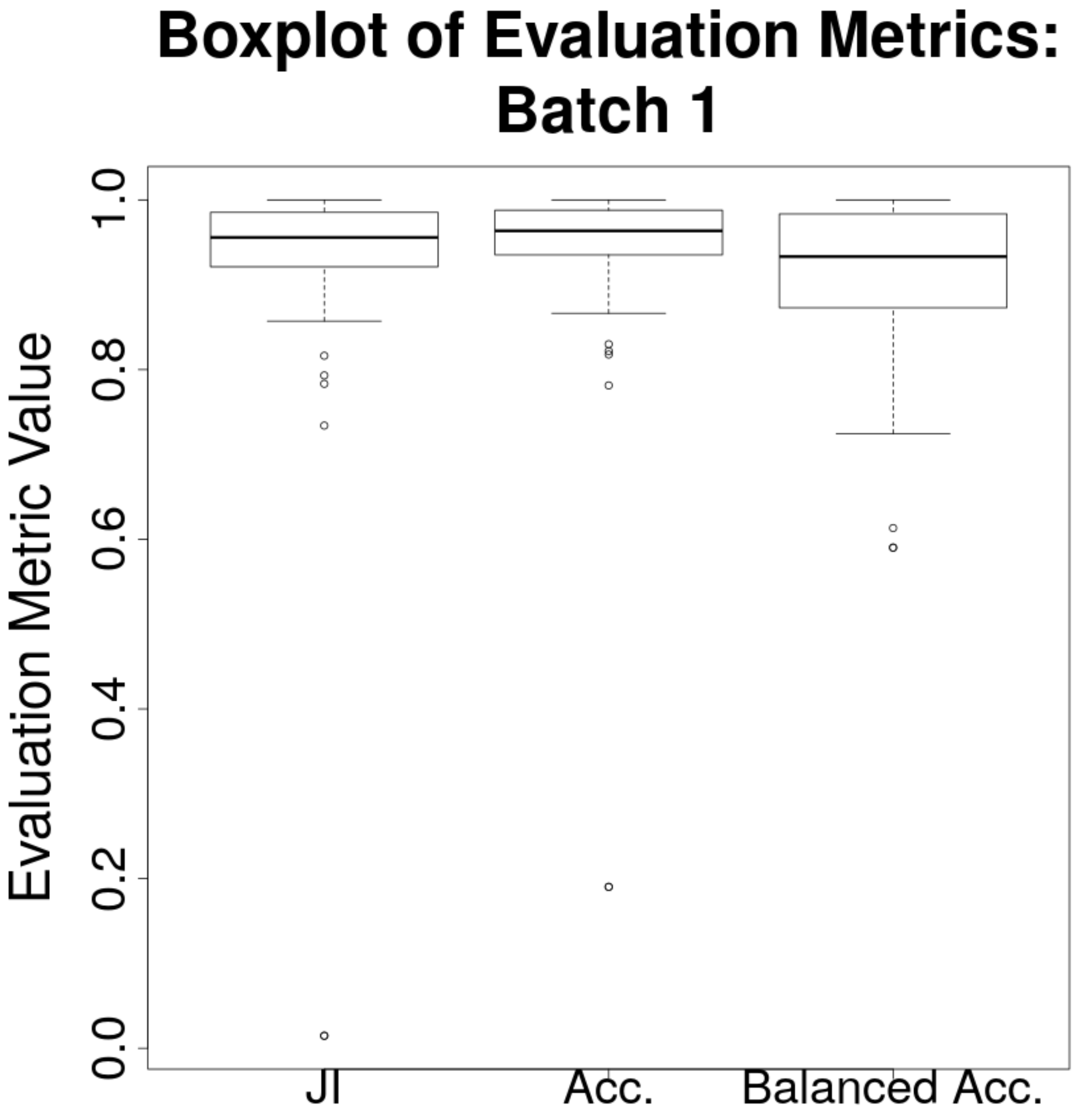


Figure B17: Boxplots of Jaccard Index (JI), accuracy (Acc.), and balanced accuracy for the first batch of the 3D-EMISH data.

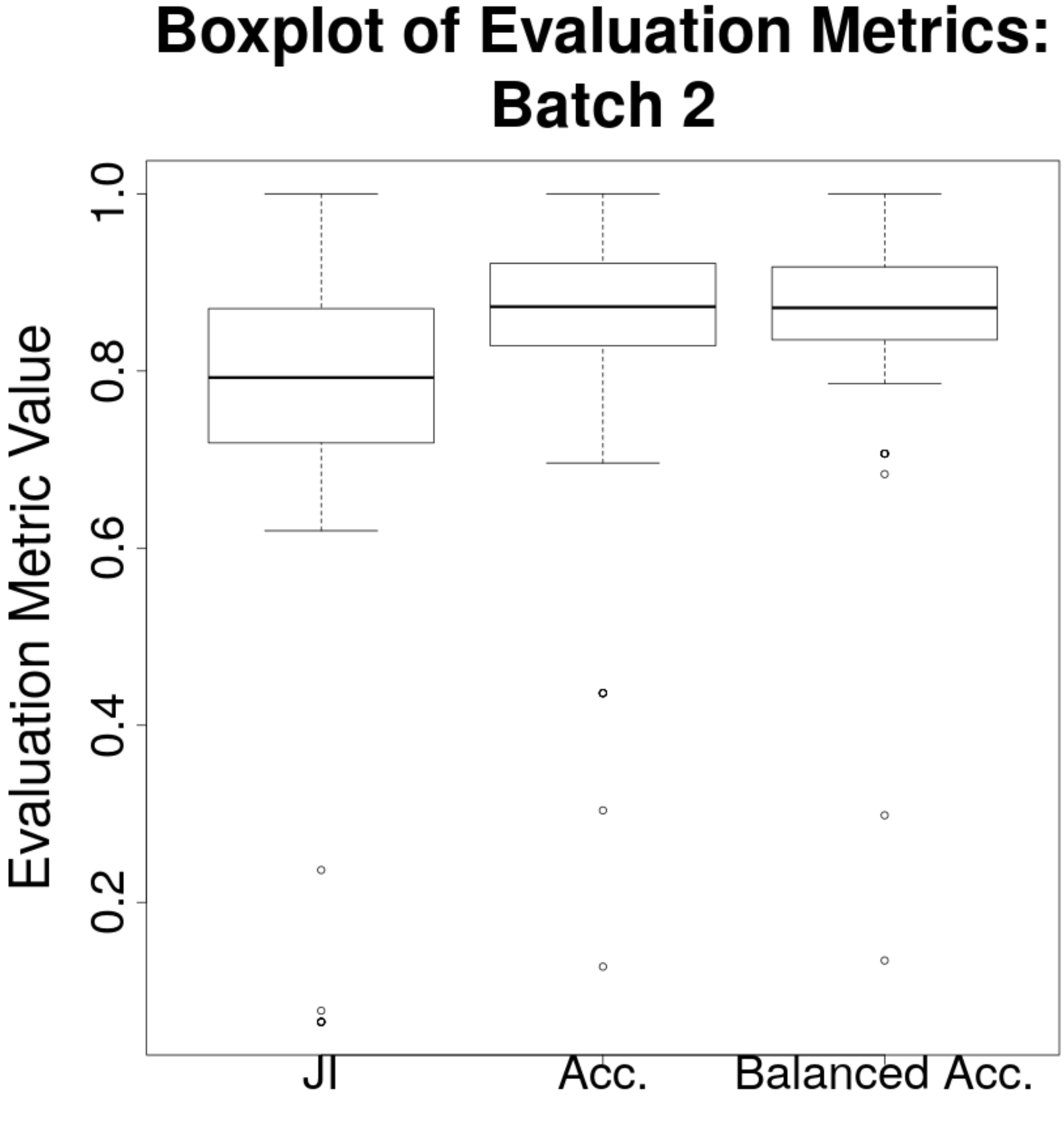


Figure B18: Boxplots of Jaccard Index (JI), accuracy (Acc.), and balanced accuracy for the second batch of the 3D-EMISH data.

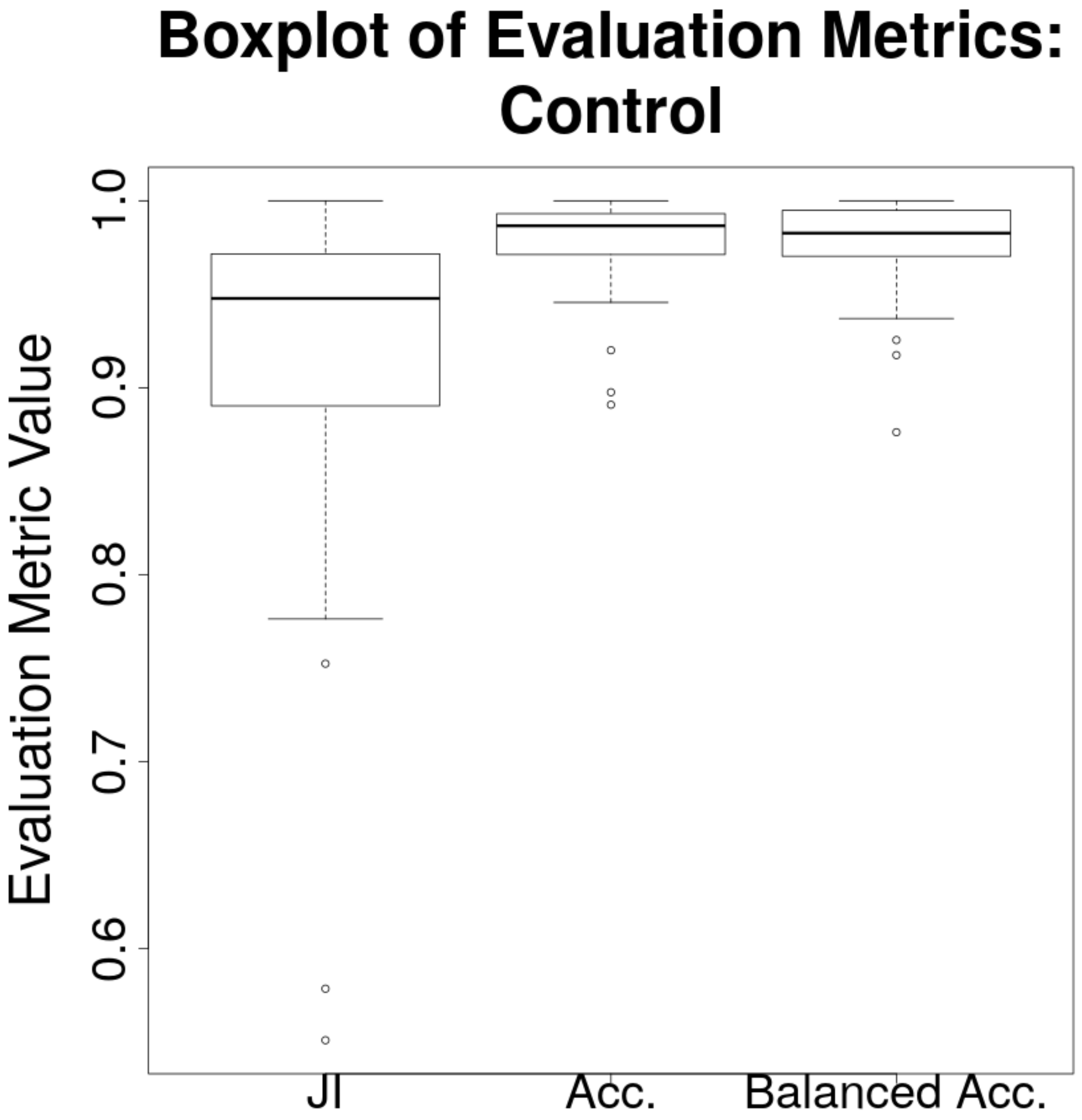


Figure B19: Boxplots of Jaccard Index (JI), accuracy (Acc.), and balanced accuracy for the control treatment of the 3D-SIM data.

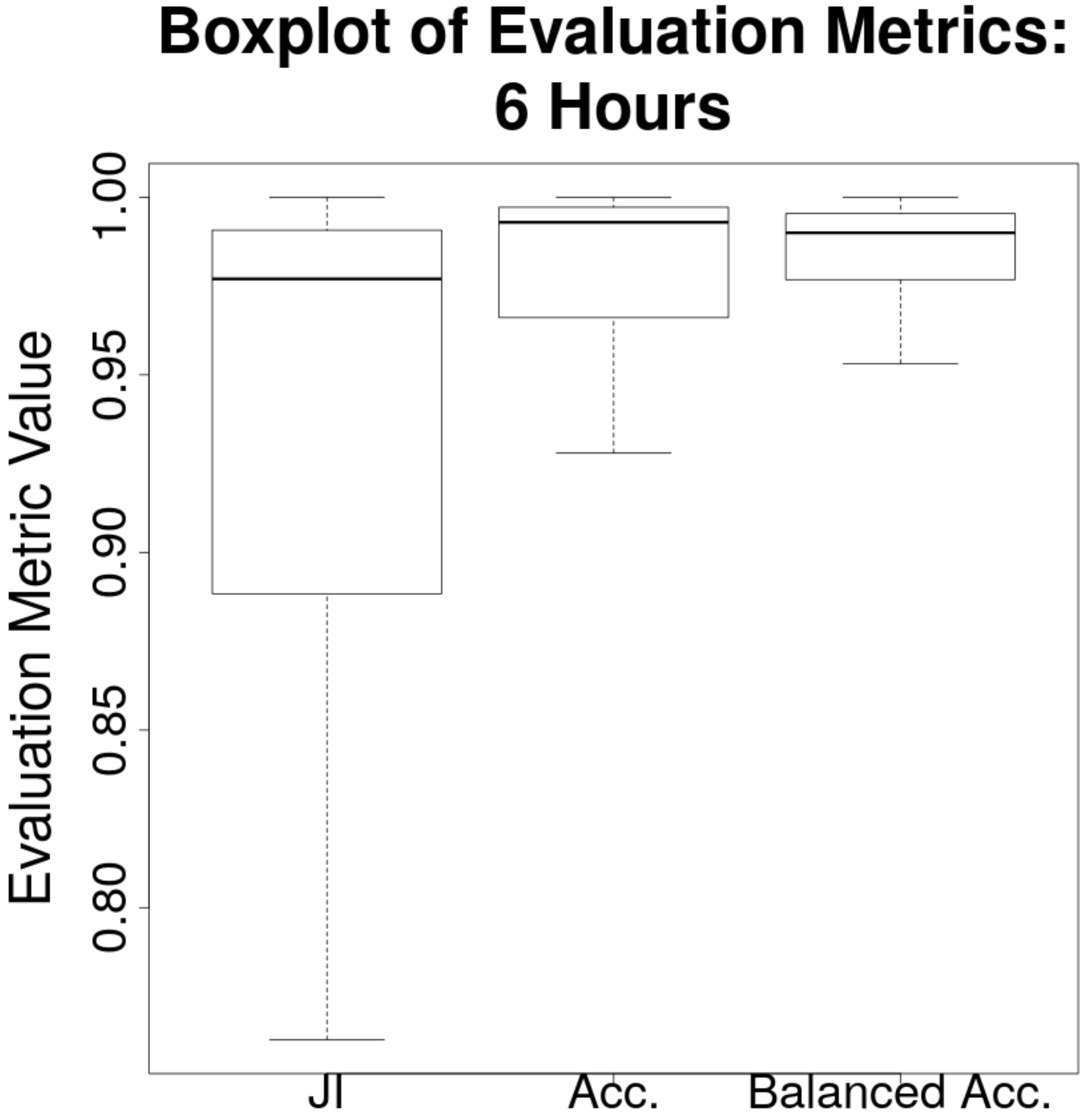


Figure B20: Boxplots of Jaccard Index (JI), accuracy (Acc.), and balanced accuracy for the 6 hour treatment of the 3D-SIM data.

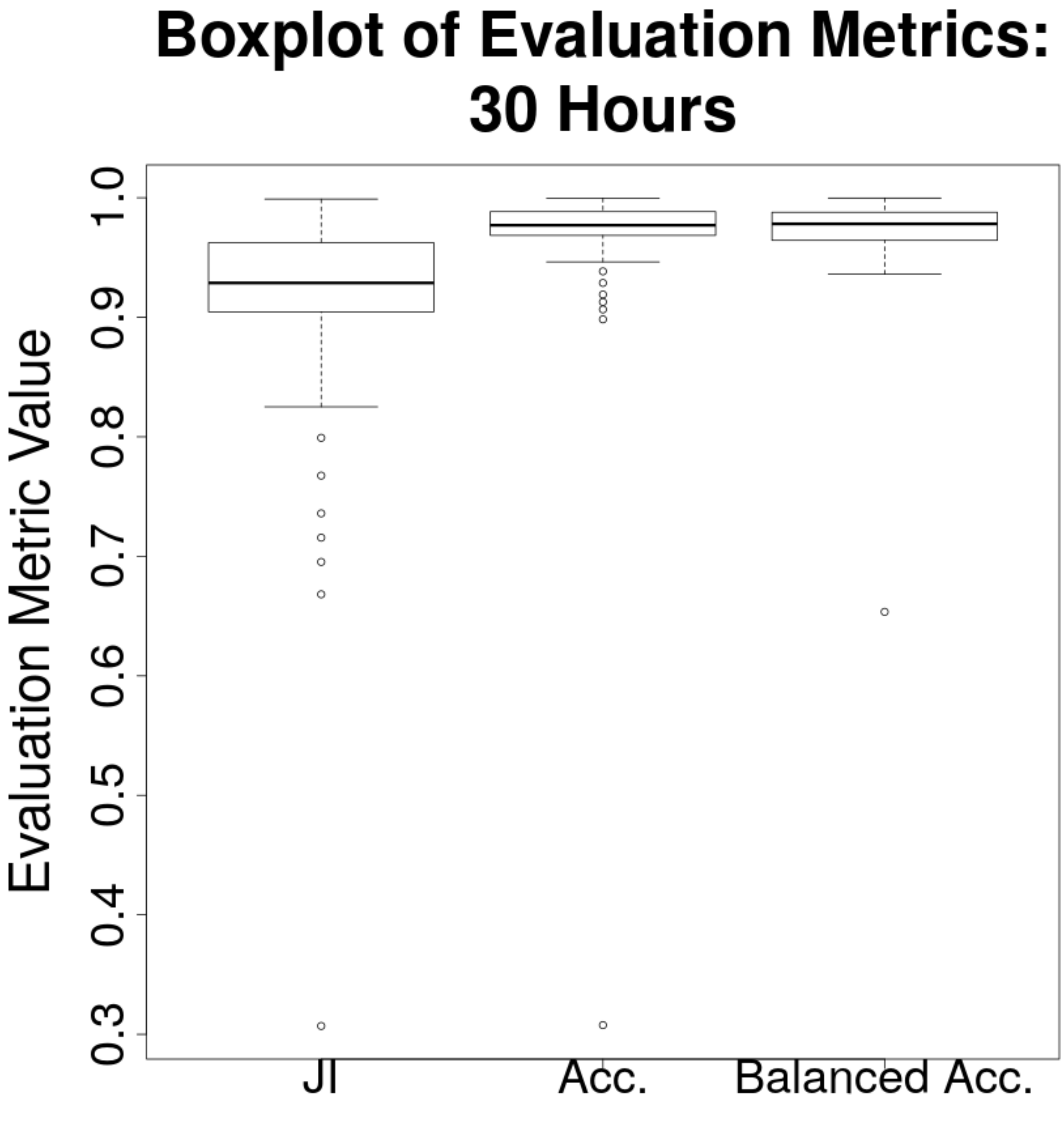


Figure B21: Boxplots of Jaccard Index (JI), accuracy (Acc.), and balanced accuracy for the 30 hour treatment of the 3D-SIM data.

# C Supplementary Tables

Table C1: The mean Jaccard Index (JI), accuracy (Acc.), and balanced Acc. for the bootstrapped clustering labels for the 3D-EMISH data across the two replicates.

| Replicate | JI | Acc. | Balanced Acc. |
|---|---|---|---|
| 1 | 0.93 | 0.94 | 0.92 |
| 2 | 0.74 | 0.84 | 0.86 |

Table C2: The variance Jaccard Index (JI), accuracy (Acc.), and balanced Acc. for the bootstrapped clustering labels for the 3D-EMISH data across the two replicates.

| Replicate | JI | Acc. | Balanced Acc. |
|---|---|---|---|
| 1 | 0.14 | 0.12 | 0.09 |
| 2 | 0.23 | 0.16 | 0.12 |

Table C3: Table shows the relative variable importance (VI) of the classification model for open and closed CDs from the 3D-EMISH data.

| Variable | Relative VI |
|---|---|
| $3^{rd}$ Intensity Quantile (Q3) | 1.0000 |
| Max Intensity (Max) | 0.8902 |
| Sphericity | 0.4891 |
| Minor Axis Length (E3) | 0.3465 |
| Major Axis Length (E1) | 0.2969 |

Table C4: Confusion table on the training 3D-EMISH data.

| Predicted/Truth | Open | Closed |
|---|---|---|
| Open | 194 | 8 |
| Closed | 7 | 106 |

Table C5: Confusion table on the validation 3D-EMISH data.

| Predicted/Truth | Open | Closed |
|---|---|---|
| Open | 83 | 2 |
| Closed | 4 | 47 |

Table C6: Table shows the relative variable importance (VI) of the classification model for open and closed CDs from the control 3D-SIM data.

| Variable | Relative VI |
|---|---|
| Max | 1.0000 |
| Surface Area | 0.8663 |
| E1 | 0.4274 |
| Min | 0.3562 |

Table C7: Table shows the relative variable importance (VI) of the classification model for open and closed CDs from the 6 hour 3D-SIM data.

| Variable | Relative VI |
|---|---|
| Surface Area | 1.0000 |
| Max | 0.7828 |
| Skew | 0.7127 |
| SD | 0.2293 |
| E1 | 0.1908 |

Table C8: Table shows the relative variable importance (VI) of the classification model for open and closed CDs from the 30 hour 3D-SIM data.

| Variable | Relative VI |
|---|---|
| Surface Area | 1.0000 |
| Max | 0.9474 |
| Skew | 0.8662 |
| SD | 0.5919 |
| Min | 0.5783 |
| E1 | 0.4860 |
| E3 | 0.3890 |

Table C9: Confusion table on the training data for the control 3D-SIM data.

| Predicted/Truth | Open | Closed |
|---|---|---|
| Open | 1340 | 5 |
| Closed | 5 | 426 |

Table C10: Confusion table on the validation data for the control for the 3D-SIM data.

| Predicted/Truth | Open | Closed |
|---|---|---|
| Open | 573 | 2 |
| Closed | 4 | 184 |

Table C11: Confusion table on the training data for the 6-hour treatment for the 3D-SIM data.

| Predicted/Truth | Open | Closed |
|---|---|---|
| Open | 338 | 0 |
| Closed | 0 | 156 |

Table C12: Confusion table on the validation data for the 6-hour treatment for the 3D-SIM data.

| Predicted/Truth | Open | Closed |
|---|---|---|
| Open | 146 | 0 |
| Closed | 0 | 68 |

Table C13: Confusion table on the training data for the 30-hour treatment for the 3D-SIM data.

| Predicted/Truth | Open | Closed |
|---|---|---|
| Open | 1337 | 9 |
| Closed | 9 | 666 |

Table C14: Confusion table on the validation data for the 30-hour treatment for the 3D-SIM data.

| Predicted/Truth | Open | Closed |
|---|---|---|
| Open | 574 | 5 |
| Closed | 4 | 285 |

Table C15: Accuracy and associated CI for LR predicting $k-$means labels.

| Experiment | Data | Acc. | CI |
|---|---|---|---|
| 3D-EMISH | Train. | 0.9841 | (0.9633, 0.9948) |
| 3D-EMISH | Valid. | 0.9853 | (0.9479, 0.9982) |
| 3D-SIM: 0 Hr. | Train. | 0.9944 | (0.9897, 0.9973) |
| 3D-SIM: 0 Hr. | Valid. | 0.9921 | (0.9830, 0.9971) |
| 3D-SIM: 6 Hr. | Train. | 1.0000 | (0.9926, 1.0000) |
| 3D-SIM: 6 Hr. | Valid. | 1.0000 | (0.9829, 1.0000) |
| 3D-SIM: 30 Hr. | Train | 0.9911 | (0.9860, 0.9947) |
| 3D-SIM: 30 Hr. | Valid. | 0.9896 | (0.9804, 0.9952) |

Table C16: The mean Jaccard Index (JI), accuracy (Acc.), and balanced Acc. for the bootstrapped clustering labels for the 3D-SIM data.

| Treatment | JI | Acc. | Balanced Acc. |
|---|---|---|---|
| Control | 0.93 | 0.98 | 0.98 |
| 6 Hours | 0.94 | 0.98 | 0.99 |
| 30 Hours | 0.91 | 0.97 | 0.97 |

Table C17: The variance Jaccard Index (JI), accuracy (Acc.), and balanced Acc. for the bootstrapped clustering labels for the 3D-SIM data.

| Treatment | JI | Acc. | Balanced Acc. |
|---|---|---|---|
| Control | 0.08 | 0.02 | 0.02 |
| 6 Hours | 0.07 | 0.02 | 0.01 |
| 30 Hours | 0.09 | 0.07 | 0.04 |

Table C18: Summary statistics for the coefficient values for bootstrapped samples for the control 3D-SIM data. Bolded text for the coefficient names indicate that the 95% inner quantile interval includes the coefficient value from Table 1.

| Coefficient | Mean | 2.5% Quantile | 97.5% Quantile |
|---|---|---|---|
| **Intercept** | -86.36 | -121.14 | -57.71 |
| **1$^{st}$Eigen.** | 0.12 | 0.01 | 0.26 |
| **S.A.** | 0.07 | 0.04 | 0.10 |
| **Max** | 0.07 | 0.04 | 0.10 |
| **Min** | -0.01 | -0.03 | 0.00 |

Table C19: Summary statistics for the coefficient values for bootstrapped samples for the 6 hour 3D-SIM data. Bolded text for the coefficient names indicate that the 95% inner quantile interval includes the coefficient value from Table 1.

| Coefficient | Mean | 2.5% Quantile | 97.5% Quantile |
|---|---|---|---|
| **Intercept** | -5891.49 | -49106.73 | -91.83 |
| **1$^{st}$Eigen.** | 36.36 | -1.02 | 353.18 |
| **S.A.** | 49.75 | 0.65 | 417.74 |
| **SD** | 6.50 | 0.06 | 61.11 |
| **Max** | 0.63 | 0.01 | 4.29 |
| **Skewness** | 142.01 | -10.63 | 1208.63 |

Table C20: Summary statistics for the coefficient values for bootstrapped samples for the 30 hour 3D-SIM data. Bolded text for the coefficient names indicate that the 95% inner quantile interval includes the coefficient value from Table 1.

| Coefficient | Mean | 2.5% Quantile | 97.5% Quantile |
|---|---|---|---|
| **Intercept** | -112.90 | -179.09 | -67.17 |
| **1$^{st}$Eigen.** | 1.36 | 0.08 | 2.95 |
| **3$^{rd}$Eigen.** | 13.48 | -2.18 | 28.57 |
| **S.A.** | 0.56 | 0.31 | 0.91 |
| SD | 0.08 | 0.05 | 0.13 |
| **Max** | 0.01 | 0.00 | 0.03 |
| Min | -0.00 | -0.01 | 0.01 |
| Skewness | 1.23 | -4.21 | 6.14 |

Table C21: Summary statistics for the coefficient values for bootstrapped samples for the 6 hour 3D-SIM data when only the top 3 variables are used. Bolded text for the coefficient names indicate that the 95% inner quantile interval includes the Estimate value. The following symbol provided next to the coefficient value indicate different levels of significance: ***=0.001, **=0.01, *=0.05, .=0.1.

| Coefficient | Estimate | Mean | 2.5% Quantile | Median | 97.5% Quantile |
|---|---|---|---|---|---|
| **Intercept** | $-166.32^{*}$ | -93.04 | -194.80 | -83.59 | -58.73 |
| **S.A.** | $1.27^{*}$ | 0.78 | 0.47 | 0.75 | 1.23 |
| **Max** | $0.04^{*}$ | 0.02 | 0.01 | 0.02 | 0.05 |
| Skewness | $8.73^{*}$ | -1.79 | -8.21 | -0.85 | 2.34 |
